# The molecular landscape and microenvironment of salivary duct carcinoma reveal new therapeutic opportunities

**DOI:** 10.7150/thno.42986

**Published:** 2020-03-15

**Authors:** Melissa Alame, Emmanuel Cornillot, Valère Cacheux, Guillaume Tosato, Marion Four, Laura De Oliveira, Stéphanie Gofflot, Philippe Delvenne, Evgenia Turtoi, Simon Cabello-Aguilar, Masahiko Nishiyama, Andrei Turtoi, Valérie Costes-Martineau, Jacques Colinge

**Affiliations:** 1 Institut de Recherche en Cancérologie de Montpellier (IRCM), INSERM, Parc Euromédecine, 208 rue des Apothicaires, 34298 Montpellier, France; 2 Biological Hematology Department, CHU Montpellier, Hôpital Saint Eloi, 34275 Montpellier, France; 3 Université de Montpellier, Faculté de Pharmacie, 15 avenue Charles Flahault, 34093 Montpellier, France; 4 Institut Régional du Cancer Montpellier (ICM), Parc Euromédecine, 208 rue des Apothicaires, 34298 Montpellier, France; 5 Université de Montpellier, Faculté de Médecine, 2 rue école de Médecine, 34060 Montpellier, France; 6 Biopathology Department, CHU Montpellier, Hôpital Gui De Chauliac, 34000 Montpellier, France; 7 Biothèque, Université de Liège, 4000 Liège, Belgium; 8 Pathology Department, CHU Liège, Université de Liège, 4000 Liège, Belgium; 9 Université de Montpellier, 163 rue Auguste Broussonnet, 34090 Montpellier, France; 10 Department of Molecular Pharmacology and Oncology, Gunma University Graduate School of Medicine, Gunma, Japan

**Keywords:** salivary duct carcinoma, stroma, personalized medicine, immunotherapy, molecular pathways

## Abstract

**Purpose**: Salivary duct carcinoma (SDC) is a rare and aggressive salivary gland cancer subtype with poor prognosis. The mutational landscape of SDC has already been the object of several studies, however little is known regarding the functional genomics and the tumor microenvironment despite their importance in oncology. Our investigation aimed at describing both the functional genomics of SDC and the SDC microenvironment, along with their clinical relevance.

**Methods**: RNA-sequencing (24 tumors), proteomics (17 tumors), immunohistochemistry (22 tumors), and multiplexed immunofluorescence (3 tumors) data were obtained from three different patient cohorts and analyzed by digital imaging and bioinformatics. Adjacent non-tumoral tissue from patients in two cohorts were used in transcriptomic and proteomic analyses.

**Results**: Transcriptomic and proteomic data revealed the importance of Notch, TGF-β, and interferon-γ signaling for all SDCs. We confirmed an overall strong desmoplastic reaction by measuring α-SMA abundance, the level of which was associated with recurrence-free survival (RFS). Two distinct immune phenotypes were observed: immune-poor SDCs (36%) and immune-infiltrated SDCs (64%). Advanced bioinformatics analysis of the transcriptomic data suggested 72 ligand-receptor interactions occurred in the microenvironment and correlated with the immune phenotype. Among these interactions, three immune checkpoints were validated by immunofluorescence, including CTLA-4/DC86 and TIM-3/galectin-9 interactions, previously unidentified in SDC. Immunofluorescence analysis also confirmed an important immunosuppressive role of macrophages and NK cells, also supported by the transcriptomic data.

**Conclusions**: Together our data significantly increase the understanding of SDC biology and open new perspectives for SDC tumor treatment. Before applying immunotherapy, patient stratification according to the immune infiltrate should be taken into account. Immune-infiltrated SDC could benefit from immune checkpoint-targeting therapy, with novel options such as anti-CTLA-4. Macrophages or NK cells could also be targeted. The dense stroma, i.e., fibroblasts or hyaluronic acid, may also be the focus for immune-poor SDC therapies, e.g. in combination with Notch or TGF-β inhibitors, or molecules targeting SDC mutations.

## Introduction

According to the World Health Organization (WHO) classification 1, salivary gland tumors constitute a heterogeneous group of tumors comprised of 24 histotypes. Among these, salivary duct carcinoma (SDC) represents 2% of primary epithelial salivary gland tumors. It is a rare cancer that predominantly arises at the parotid or submandibular region, and is characterized by the degree of aggressiveness and high mortality rate. At diagnosis, patients often present lymph node involvement. Standard therapy primarily relies on local resection with adjuvant radiotherapy. Nevertheless, recurrence and distant metastases frequently occur. These respond poorly to chemotherapy, this being the common second-line treatment 2. Given the known androgen receptor (AR)-positive and human epidermal growth factor receptor 2 (HER2)-positive histological status of SDC, WHO classifies SDC neoplastic tissue as similar to invasive ductal mammary carcinoma (IDC) 1. Initial studies have reported potential efficacy of androgen deprivation therapy (ADT) 3 or HER2 inhibition, *e.g.*, with trastuzumab 4. Unfortunately, no pre-clinical models of SDC are available to date.

Significant efforts have been devoted to the investigation of somatic mutations in SDC and into the identification of genetic insults that could be targeted 5-11. These studies revealed mutations in cancer genes involved in, for example, DNA damage, mitogen-activated protein kinase signaling, receptor tyrosine kinase signaling, PI3K-AKT signaling, androgen signaling, and histone modifiers. Recurrent- mutated genes are *TP53* (varying among reports: 31%- 68% of patients), *PIK3CA* (9%-37% of patients), *HRAS* (11%-27% of patients)*, FOXA1* (0%-25% of patients) and *NF1* (0%-18% of patients). Some mutations may limit the benefit of ADT, *e.g.*, *FOXA1* 5. Gene copy number alteration (CNA) analysis has indicated a modest rate of chromosome arm amplifications or deletions 5,8, and a limited number of gene fusion events have been identified 5. The transcriptomes of 16 SDCs were obtained and analyzed, showing transcriptional resemblance with breast tumors 5. To date, however, no comparison of SDC *versus* normal adjacent salivary duct tissue has been conducted to discover SDC-deregulated pathways.

The contribution of the tumor microenvironment (TME) to tumor progression and therapy resistance is substantial in most solid tumors 12. Immunotherapies have revolutionized the treatment of cancer, and antibodies targeting immune checkpoints or ligands, *e.g.*, PD-1/PD-L1 or CTLA-4, have demonstrated clinical benefit. In particular, anti-PD-1/PD-L1 in head and neck or salivary gland tumors has shown potential 13-15. Given the immune-expression of PD-1, PD-L1, major histocompatibility complex class I (MHC I), and the cancer-testis antigen PRAME in various cancers, a recent study probed the TME of 53 SDCs 16. The investigators determined a correlation between the expression of PD-1, PD-L1, and PRAME. In addition, the expressions of PD-1 in immune cells, and PD-L1 in tumor cells, were significantly associated with patient survival. Additionally, MHC I downregulation was observed in 82% of the SDCs. Taken together, these results indicate a potential for therapies targeting the TME. Additional properties of the TME, *e.g.*, a desmoplastic stroma or immunosuppressive elements, may nonetheless limit the benefit of immunotherapies and should be investigated in detail.

In this study, a comprehensive functional genomic characterization of SDC was carried out on three different patient cohorts, including both tumoral and non-tumoral tissues. Transcriptomic and proteomic analyses were exploited to unravel important molecular pathways that are involved in this cancer. In particular, several pathways related to extracellular matrix (ECM) remodeling, inflammation, and immunosuppression were apparent and have potential implications in personalized patient therapy. Based on genomic, immunohistochemistry (IHC), and multiplexed immunofluorescence (IF) data, examination of the SDC TME composition enabled the definition of two main SDC subtypes: immune-poor and immune-infiltrated SDCs. In addition, we revealed the importance of macrophages in SDC and highlighted several potential targets for SDC microenvironment disruption.

## Materials and Methods

Additional details on the patient cohorts, proteomics, IHC, and multiplexed IF are provided as Supplementary Material.

### Patients and cohorts

Published data for 16 SDC patients, referred to as the MSKCC cohort (see the original publication for details 5), were combined with data from 20 additional patients from France and 4 from Belgium (**Table 1**). The patients diagnosed with SDC provided written informed consent for tissue collection and subsequent research. For the first cohort of 8 French patients (cohort 1), tissues from FFPE blocks plus snap-frozen material obtained at the time of surgery were available. Normal salivary gland tissues from two patients were also collected. For the second cohort of 16 French and Belgian patients (cohort 2), only FFPE blocks were available. In addition, normal salivary gland tissue from three patients were provided; plus, for one patient, tissues from metastatic and primary SDCs were available.

### Transcriptomics

RNA was extracted from cohort 1 samples and quality-controlled. DNA libraries were prepared with the NEBNext Ultra II mRNA-Seq kit and sequenced on a HiSeq4000 (Illumina) using 2×75pb cycles to generate 130 million reads. MSKCC cohort transcriptomes (Fastq files) were downloaded from the NCBI Sequence Read Archive. With our pipeline, Fastq files of cohorts 1 and MSKCC were aligned against the human genome (Ensembl GRCh38) (STAR using default parameters and 2 passes, read counts extraction with HTSeq-count). See **Supplementary Material, Table S1**, and **Figure S1** for batch-effect correction and data normalization. Data are accessible from GEO (GSE138581).

### Proteomics

Twenty FFPE tissue sections (5 µm thickness) were deparaffinized and the proteins digested by trypsin. Following digestion, 10% of each sample was mixed to create a pool for generation of the spectral library. The samples were analyzed on a nano-HPLC system (Sciex, Framingham, MA, USA) connected on-line to an electrospray Q-TOF 6600 mass spectrometer (Sciex). Two acquisition modes were used: data-dependent (DDA) to generate the reference spectral library, and data-independent (DIA or SWATH) to measure the samples. The library data were searched against the human protein database using Protein Pilot (Sciex). For the SWATH acquisition, the DDA method was adapted using the automated method generator embedded in the Analyst software (Sciex). Protein identification and quantitation were conducted with the Peak View software and the previously-generated protein library. Expression data for 3,203 proteins were obtained, and normalized against the total ion intensity before log_10_-transformation. Data are accessible from PRIDE (PXD015885).

### Differential gene and protein analyses

Differential gene analysis was performed with edgeR 17, P-value < 0.01, FDR < 0.01, minimum fold-change 2, and a minimum of 20 (normalized) read counts over all the samples analyzed. Differential protein expression was performed with limma 18, P-value < 0.05, minimum fold-change 1.25, and a minimum average (log_10_-transformed) signal of 3. Heat maps were generated with ComplexHeatmap 19. Dendrograms that ordered samples (columns) and genes/proteins (rows) were constructed with Ward's method based on the Euclidean distance. For color assignment, a threshold of 2.5% was applied to the upper and lower values.

In conjunction with a hypergeometric test, GO terms and Reactome pathways were used. This was followed by Benjamini-Hochberg multiple hypothesis correction (internally-developed R script), FDR < 0.05 and a minimum of 5 query genes/proteins in a pathway or GO term.

### Immunohistochemistry

Paraffin sections (5 µm thick) were deparaffinized. Antigen retrieval was performed using AR6 buffer for 10 min in a pressure cooker. The sections were blocked for 30 min in protein block serum-free solution and incubated with the primary antibody at room temperature (RT) for 2h (see **Table S2** for antibodies used). The slides were then washed and incubated for 30 min at RT with the secondary antibody. Subsequently, the sections were washed and then stained with 3,3'-diaminobenzidine (DAB). Stained sections were imaged with an automated Nanozoomer 2.0HT (Hamamatsu) at ×40 magnification (230 nm/pixel).

DAB-positive stained cells (CD3 and CD8 markers) were automatically counted using the open-source software Qupath 20. Due to particular cell shapes, macrophages (CD68, CD163) and α-SMA markers were evaluated by counting DAB positive pixels to improve accuracy. Intensity thresholds and other parameters for cell/pixel detection and classification were manually set for each staining type and performed identically for all samples. A machine learning-based method was applied to annotate stromal area and tumor nests within the tumor core. For further analyses, cell and pixel densities were estimated as the percentage of positive cells per mm² and the percentage of positive pixel per mm² of surface area, respectively 20. All steps were performed under the supervision of an expert pathologist (VCM). Necrosis, tissue folds and entrapped normal structures were carefully removed.

### Multiplexed Immunofluorescence

Tissue sections were prepared as described above with the exception that incubation with the primary antibody that was conducted at 4°C overnight and the staining was performed using the Opal system (Perkin Elmer). Following primary antibody incubation, the slides were incubated with the corresponding secondary antibody as described above. The slides were then incubated with 100 μL staining solution prepared from 2 μL Opal dye and 98 μL amplifying buffer. Following 10 min incubation, the slides were washed and subjected to microwave-assisted antibody removal. After cooling and a wash in PBS buffer for 5 min, the tissues were re-blocked. Tissues were then incubated with the next primary antibody and the staining procedure was repeated using the following Opal dyes: 520, 570, 620 and 690.

Multiplexed immunofluorescence (IF) images were treated with Fiji software and analyzed with an internally-developed R script. The images were converted to an 8-bit grayscale; a lower threshold was applied to remove the background noise and an upper threshold was applied to rescale the maximum gray value. The IF image of the receptor was binarized to isolate the cells expressing the receptor. The *Analyze Particles* plugin of Fiji was used to locate the cells and extract the positions of the centroid. Only the shapes with a circularity > 0.3 and an area > 50 pixel² were retained. The R script used these positions to calculate the average fluorescence of the receptor inside a circle with a diameter equal to a receptor-expressing cell and the average fluorescence of the ligand inside a surrounding crown (**Figure S2A**). The averaged fluorescence values were then used to calculate an IF ligand-receptor score (ifLR-score). A threshold on the ifLR-score was determined to assess if the interaction was positive for each cell expressing the receptor (**Figure S2B**). See Supplementary Materials for details of the score calculation and threshold adjustment.

### Ligand-receptor interactions and pathways

Interactions from FANTOM5, HPRD, HPMR, the IUPHAR/BPS Guide to pharmacology, UniprotKB/ Swissprot annotations, Reactome, plus manual extraction from cellsignaling.com maps and the literature were combined. Reactome-derived ligand-receptor (LR) pairs corresponded to protein interactions from Reactome with the respective participants annotated as ligand or receptor in Gene Ontology (GO). Reactome pathways that were downloaded as a collection of binary interactions from PathwayCommons, were also used. For a few receptors that remained unconnected in Reactome, these were manually complemented with interactions annotated in UniprotKB/Swissprot (**Table S3**).

Confident LR interactions that occur in SDC were determined by firstly imposing Spearman r > 0.5 between a ligand and its receptor and Benjamini- Hochberg corrected P-values of these correlations < 0.01. This resulted in 277 LR pairs. Receptor downstream activity was then assessed by considering all Reactome pathways containing the receptor. In each pathway, the target genes were identified plus otherwise controlled genes, *e.g.*, by phosphorylation of the product, and the criterion that at least 4 displaying Spearman r > 0.5 with the receptor was imposed. If only 2 or 3 targets/controlled genes were available, r > 0.5 was required for all. This procedure selected 151 confident LR pairs. The same algorithm was applied for GO biological process (GOBP) terms as *ad hoc* pathways. Their topology was retrieved from Reactome interactions and 144 confidence LR pairs were obtained. In total, 179 unique confidence LR pairs were determined.

## Results

### Functional genomics of salivary duct carcinoma

**Figure 1:**
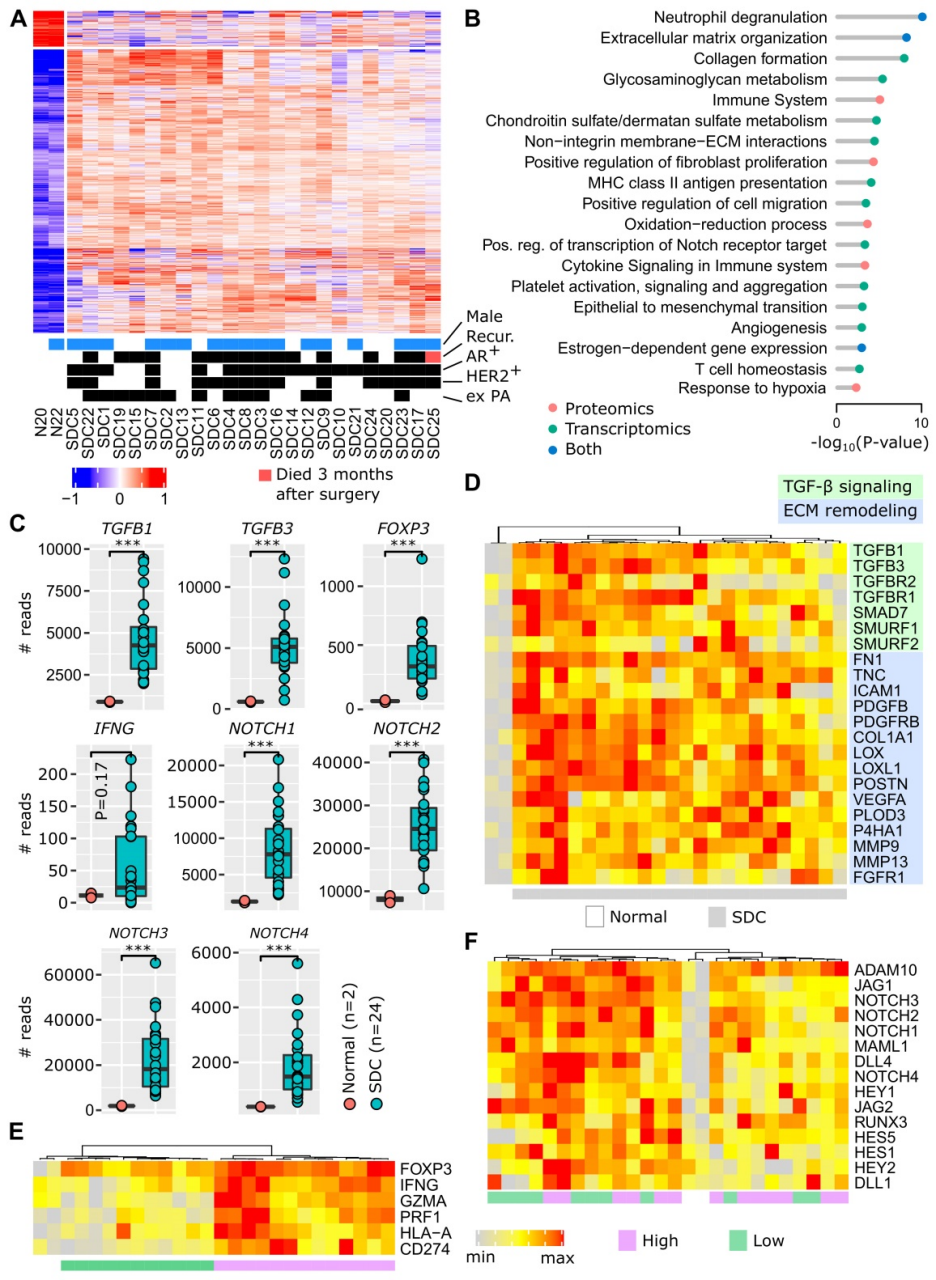
** Functional genomics.** (A) Differentially-expressed genes between adjacent normal tissues and SDC. (B) Selected pathways. (C) Expression of important genes (Wilcoxon one-sided test, n=26=2+24, *** P<0.005). (D) TGF-β and ECM remodeling genes. (E) Inflammation and immunosuppression genes. Two clusters of tumors exist, denoted high and low. The expression of *FOXP3* is high in all SDCs. (F) Notch signaling genes. Note that the gradient (from left to right) of Notch signaling gene expression is not correlated with clusters in panel F.

The access to non-tumoral tissues in both our patient cohorts (cohorts 1 and 2) enabled us to ask the fundamental question: which pathways are deregulated in SDC and can any of them be targeted? This investigation was initiated at the transcriptional level by combining the RNA-seq data from the MSKCC cohort and our cohort 1 (8 new French patients). The merged data sets were submitted to the same bioinformatics pipeline and SDC* versus* non-tumoral tissues were compared. This revealed that 1,634 genes were significantly deregulated (**Figure 1A**), the majority (1,451) with increased expression in SDCs. Similarly, our cohort 2 (16 French and Belgian patients) was investigated to perform a similar comparison at the protein level. This resulted in 244 significantly deregulated proteins, 213 with increased expression (**Figure S3**) in SDCs. Among the 244 deregulated proteins, 85 were also deregulated at the gene level (P <10^-27^, hypergeometric test). Pathway enrichment analysis was conducted separately on the deregulated genes and proteins. Beyond known pathways commonly deregulated in tumors (transcription, cell cycle, *etc.*), a number of more SDC-relevant pathways were selected. These are featured in **Figure 1B** (complete lists in **Tables S4** and **S5**).

The invasive component of SDC presents as a desmoplastic stromal reaction (DSR) with a partially-hyalinized ECM. This is consistent with ECM remodeling, collagen formation, glycosaminoglycan and chondroitin metabolism, integrin interactions, and increased fibroblast proliferation pathways. ECM remodeling contributes to fibrosis, tumor stiffness, conditions for a neo-angiogenesis- supportive environment and tumor cell spreading. Several additional deregulated pathways indicated that these processes occur in SDC: epithelial to mesenchymal transition (EMT), angiogenesis, and hypoxia. Of particular interest, TGF-β expression was strongly augmented (**Figure 1C**), and this is already known to contribute to an immunosuppressive TME 21,22. In **Figure 1D**, the activation of TGF-β signaling is illustrated, including the activity of several genes involved in ECM remodeling. Depending on individual SDCs, we observed varying degrees of gene expression. However, all genes were clearly overexpressed in SDCs compared to non-tumoral tissues.

**Figure 1B** shows an increased expression of pathways related to the inflammatory response (neutrophil degranulation, MHC class II antigen presentation, cytokine signaling), together with immunosuppression (T cell homeostasis). This is supported by *FOXP3* upregulation (**Figure 1C**), a transcription factor expressed by regulatory T cells (Tregs). An increase in interferon-γ (*IFNG*), a cytokine that can activate macrophages and NK cells, is also apparent. Interferon-γ adopts a pro-tumoral and immunosuppressive role in certain tumors 23. Within the limits of our small cohort 1, a significant association of *IFNG* levels with relapse was indeed observed (**Figure S4**). Representative genes for inflammation and immunosuppression are featured in **Figure 1E**, *e.g.*, T cell effectors, such as perforin (*PRF1*) and granzyme A (*GZMA*), or the immune checkpoint ligand PD-L1 (*CD274*). Two groups of SDC are put forward by the gene expression pattern: high *versus* low level of inflammation/immunosuppression. For each SDC, ubiquitous* FOXP3* expression is consistent with TGF-β signaling.

Notch family members appear to be important in SDC (**Figure 1C**), and many downstream genes of the Notch signaling pathway were overexpressed (**Figure 1F**). Notch contributes to cancer cell development, but also to the dialog and interaction with the TME 24. A gradient of Notch signaling intensity can be seen across the SDCs in **Figure 1F**.

Activation of estrogen-dependent gene expression correlates with the apocrine-like nature of SDC transcriptomes 5 and the frequent AR-positive histological status (~80%). Contrary to breast cancer, clear and coherent transcriptional subtypes were not observed (**Figure 1A** and **Figure S3**). Correlation of protein and gene expression with clinical data (recurrence, HER2 or AR-positive staining, ex pleomorphic adenoma (PA) origin) was attempted. However, no significant or biologically sound association was observed.

### Probing the microenvironment of salivary duct carcinomas

**Figure 2:**
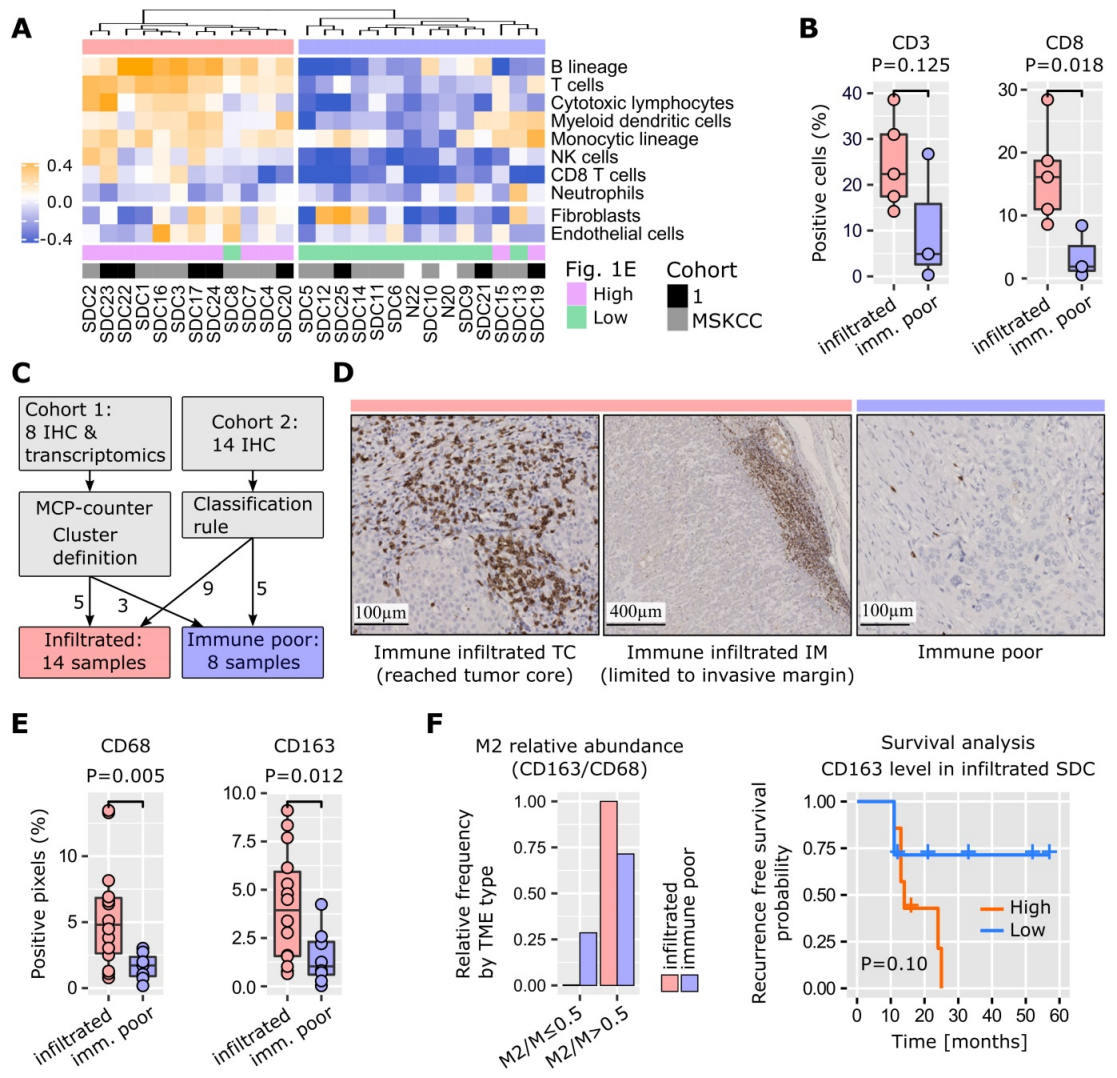
** TME cell types.** (A) Application of MCP-counter to SDC transcriptomes revealed two groups of SDC: immune-infiltrated (light red) and immune-poor (light blue). Note that these two groups were almost identical to the high/low clusters in Fig. 1E. (B) Validation of the differential T cell infiltrates between the groups (Wilcoxon one-sided tests, n=8=5+3). (C) Samples from cohort 2 that were not available for transcriptomics were added to the IHC study to obtain 22 SDCs. Samples were classified according to CD3+ and CD8+ cell abundance and localization. (D) Two CD3+/CD8+ patterns were observed with the immune-infiltrated group: limited to the IM, or present in the TC. (E) Total macrophages (CD68) and M2 (CD163) were more abundant in the immune-infiltrated SDC (specific pattern ignored), (Wilcoxon one-sided test, n=21=14+7 for CD68, one immune-poor outlier removed (significant for Grubbs and Dixon tests, robust (median and MAD) z-score>3); n=22=14+8 for CD163). (F) Distribution of SDC with M2 macrophages representing > 50% of the macrophages.

A software tool, MCP-counter 25, was used to identify TME cell populations from transcriptomic data. Due to the modest size of our cohort, and the histological proximity of SDC with breast IDC 1, 624 breast IDCs were retrieved from the cancer genome atlas (TCGA) and were submitted to MCP-counter together with the SDCs. This enabled better normalization of the MCP-counter scores for each TME cell type. Depending on the number of immune cells, SDCs clearly formed two distinct clusters (**Figure 2A**). Normalized fibroblast and endothelial cell abundances were represented but not used to build the dendrogram. They did not correlate with the clusters. Cluster 1 (n=12) was enriched for immune cells: T and B lymphoid cells, and monocytic lineage cells. Cluster 2 (n=14) was represented by low lymphoid cell infiltrate, but contained heterogeneous amounts of cells from the monocytic lineage. Due to clinical relevance, MCP-counter estimates were validated by IHC (using CD3 and CD8 antibodies) on the 8 SDCs from our cohort 1 (**Figure 2B**). Differential gene expression between cluster 1 and 2 confirmed a strong and almost exclusive variation in immune pathways (**Figure S5**). Hence, cluster 1 was termed *immune-infiltrated* and cluster 2, *immune-poor*. The 8 SDCs from cohort 1, together with 14 SDCs from cohort 2, for which we also performed IHC, were classified with respect to CD3- and CD8-positive cell enumeration and localization (**Figures 2C** and **S6**). In 8/22 cases (36%), a very low number of T cells were observed (**Figure 2D**); these comprised the immune- poor group. Two patterns of immune-infiltrated SDC were apparent (**Figure 2D**). In 4/22 (18%) tumors, CD8+ T cells were restricted to the invasive margin (IM). These have been previously characterized as T cell-excluded 26, and are defined here as the *immune-infiltrated IM* group. Another group of tumors, 10/22 (46%), displayed T cells either in the tumor core (TC) only, or in the TC and at the IM. We referred to this group as the *immune-infiltrated TC* group, and a quasi-exclusion of CD8+ T cells from the tumor nests in these 10 cases was observed. This particular pattern has been previously described in lung, pancreatic, and ovarian carcinomas, and was also characterized as T cell-excluded 27.

Tumor-associated macrophages (TAMs) play an important role in TME homeostasis and resistance to various treatments 26,28-30. MCP-counter analysis indicated a variable presence of TAMs in the two SDC groups (monocytic lineage in **Figure 2A**). Therefore, TAM density within the stromal areas of the TC was assessed by IHC and digital imaging. Macrophages and the alternative activated phenotype (called M2) were stained for with CD68 and CD163 respectively. A significantly higher TAM content in the immune-infiltrated SDC was observed (**Figure 2E**). The proportion of stromal M2 macrophages (M2/M ratio) was based on the CD163/CD68 density ratio. A high M2/M ratio (> 0.5) was seen in 6/8 (75%) of the SDCs with an immune-poor phenotype, and 13/13 (100%) with the immune-infiltrated phenotype. This suggested that M2 macrophages represent a significant proportion of TAMs in SDC (**Figure 2F**). With respect to M2 abundance in the TME, RFS analysis indicated a clear trend. Previous results in breast, pancreatic, and oral cancers are in agreement with this finding 29-31, thus indicating the potential relevance of TAM-targeting therapies for SDC.

**Figure 3:**
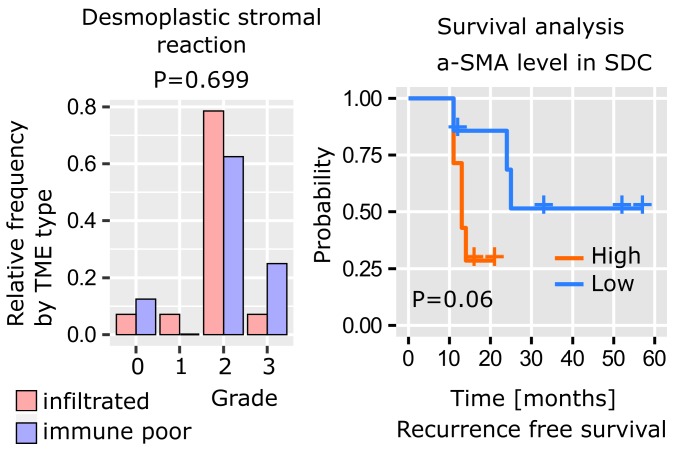
** SDC desmoplastic stromal reaction.** The distribution of DSR grades is comparable between immune-infiltrated and immune-poor SDC (Kolmogorov-Smirnov test, n=22) and recurrence-free survival (Kaplan-Meier curve, log-rank test, n=22, high=above median, low=below median).

As previously mentioned, SDC histology is characterized by a dense stroma. The desmoplastic stromal reaction (DSR) was assessed by measuring α-SMA stromal density and graded as follows: grade 0 (< 5%), 1 (5-15%), 2, (15-50%), 3 (> 50%). The data revealed that 22/29 (86%) SDCs displayed a DSR grade ≥ 2 (**Figure 3**). Interestingly, the DSR was independent of the immune infiltrate (**Figure S7**), and, according to our data there was a trend between α-SMA levels and RFS (**Figure 3**).

### Mapping cellular interactions in the SDC stroma

**Figure 4:**
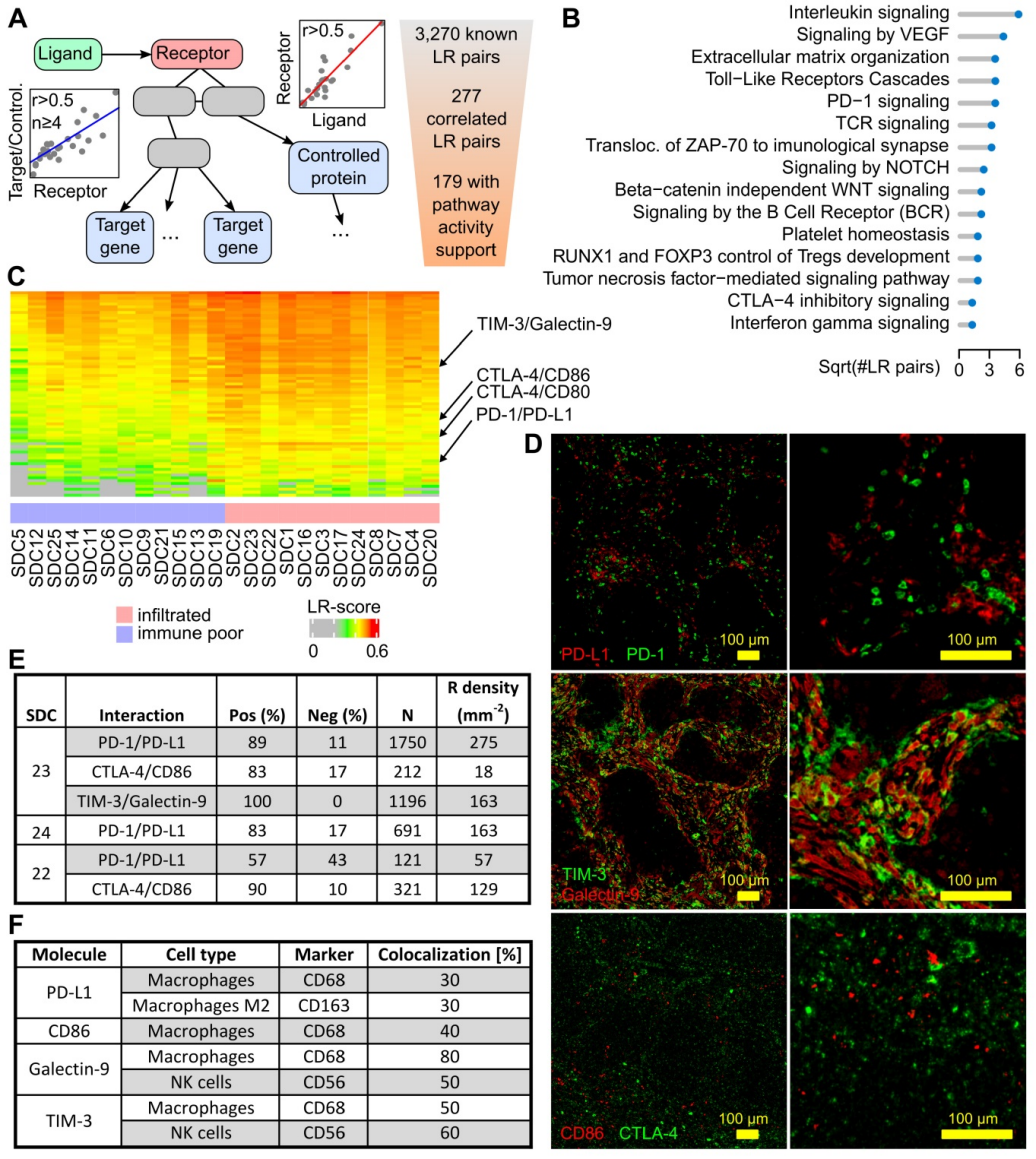
** Ligand-receptor interactions within the SDC microenvironment.** (A) Principle of the LR pair-search algorithm: the ligand and the receptor are required to correlate over the SDC transcriptomes, and the candidate receptor has to occur in pathways with at least four downstream-regulated genes (transcription factor targets) or regulated proteins (phosphorylation or other PTM) that display sufficient correlation with the latter receptor. (B) Functional categories where the ligands and the receptors occur. (C) Ligand-receptor pairs with transcriptional LR-score correlated with the immune-infiltrate category (72 pairs). (D) The three selected pairs in SDC23. (E) Ligand-receptor co-localization results. (F) Semi-quantitative assessment of the degree of fluorescence for the ligand (PD-L1, galectin-9, CD86) or receptor (TIM-3) colocalized with the marker fluorescence for each assessed cell type (note that for galectin-9, the fluorescence of CD56+ and CD68+ cells sums to 130%, but these measurements were obtained from different slides and are semi-quantitative only).

The intercellular communication network of a tumor is significantly rewired compared to the original healthy tissue. A typical illustration is the induction of PD-1-positive T cell inhibition and resulting functional exhaustion of PD-L1-expressing epithelial cancerous cells and/or immune-infiltrated cells 32. It is reasonable to suggest that a systematic study of cellular interactions within the SDC TME may unravel elements that can be potentially targeted. Ligand-receptor (LR) interactions from several public databases and the literature were compiled to assemble a database (LR*db*) comprised of 3,270 unique LR pairs. Next, an algorithm (**Figure 4A**) was developed to search for evidence of these interactions in SDC transcriptomes. Briefly, each LR pair in LR*db* was assessed and a Spearman correlation r > 0.5 was imposed between a ligand and its receptor, resulting in 277 filtered LR pairs. Evidence for downstream receptor activity was then assessed using Reactome pathways and additional correlations. This procedure (Materials and Methods) selected 179 confident LR pairs (**Table S6**). Our algorithm associated each receptor with pathways, the recurrent ones, summarized in **Figure 4B,** largely mirror the TME-associated pathways in **Figure 1B**.

### Targets for immunotherapy

The search for immune infiltrate-related LR pairs was achieved by computing a score (the LR-score) that reflected the co-occurrence of the ligand and the receptor in each SDC transcriptome (Materials and Methods). A Spearman r > 0.6 was imposed with MCP-counter immune cell gene signatures to select 72 LR pairs (**Figure 4C** and **Figure S8** with LR pair names). From these pairs, three immune checkpoints were chosen for validation by multiplexed IF: PD-1/PD-L1, CTLA-4/CD86, and TIM-3/galectin-9 (**Figure 4D**). The percentage of cells expressing the receptor that were in proximity to cells expressing the ligand were counted. This meant that the signal from the extracellular ligand must have overlapped with the receptor signal at the cell membrane in order to be counted as positive. This enabled the determination of average cell diameters, the definition of a crown-shaped signal overlap area, and the computation of a score in this area, *i.e.*, an analog of the LR-score (Materials and Methods).

#### PD-1/PD-L1 interaction

In three SDCs classified with the immune- infiltrated phenotype, a potential interaction between PD-1 and PD-L1+ cells was investigated. Two SDCs were from the TC group (SDC23 and SD24) and one SDC was from the IM group (SDC22). For SDC23 and SDC24, a large percentage of PD-1+ cells adjacent to PD-L1+ cells were observed: 89% and 83% respectively (**Figure 4E**). In SDC22, this percentage (57%) was lower but superior to 50%. The results indicated that a substantial proportion of PD-1+ cells were exhausted in the TME of immune-infiltrated SDCs. This may explain the absence of an association between CD8+ T cells and RFS with such tumors (P=0.94, see **Figure S9**). It was also noted that SDC22 (immune-infiltrated IM phenotype) contained less PD-1+ cells. The expression of PD-L1 by M2 (CD163) and non-M2 macrophages (**Figure 4F**) was further shown. A proportion of the fluorescence could not be attributed to macrophages and was assumed to originate from other cells.

#### CTLA-4/CD86 interaction

CTLA-4 is an immune checkpoint, and its expression in SDC has never been studied. Inhibition of CTLA-4+ cells by adjacent CD86+ cells (TAMs) was assessed in SDC23 and 22. In both cases, the percentage of positive counts was high: 83% and 90% respectively. The density of CTLA-4+ cells, however, was less than PD-1+ cells. This was particularly evident in SDC23 (**Figure 4E**). CD86 was expressed by CD68+ cells, but not only by this cell type.

#### TIM-3/Galectin-9 interaction

T-cell immunoglobulin mucin receptor 3 (TIM-3, *HAVCR2* gene) is an immune checkpoint that plays a role in T-cell exhaustion, and binding to galectin-9 (*LGALS9* gene) suppresses the T cell response. The TIM-3/galectin-9 interaction was assessed in SDC23 and 100% of cells were found to express TIM-3 adjacent to galectin-9+ cells (**Figure 4E**). Galectin-9 co-localized with CD68+ (macrophages) and CD56+ (NK) cells (**Figure 4F**). These two cell types do not account for the entire galectin-9 signal, indicating that other cells also express the ligand. Strikingly, co-localization of TIM-3 with CD68 and CD56 gave the same results, suggesting a potential (paracrine) cross-inhibition of macrophages and NK in addition to the autocrine inhibition. IF images show clear examples of each cell type expressing both galectin-9 and TIM-3 (**Figure S10**). From the literature, it is known that TIM-3 overexpression is observed in NK and macrophages in advanced tumors 33,34.

#### Additional targets

Beyond the three validated LR interactions above, several additional interactions (**Figure S8**, **Table S6**) were also supported by the literature. For example, the leukocyte immunoglobulin-like receptors *LILRB1* and* LILRB2* have been predicted to interact with HLA class I molecules, and they correlate with immune and monocytic lineage gene expression signatures in transcriptomics. Upregulation of either LILRB1 or LILRB2 in macrophages has shown an evasion mechanism for cancer cells against phagocytosis. Through activation of AKT and IL-4 signaling, LILRB2 antagonism induced a reduction in PD-L1 expression by macrophages and reprogramming of lung TAMs 45. Likewise, we observed in **Figure S8** and **Table S6** that CCR5 and ligands (CCL3, CCL4, CCL5, CCL8, CCL11, and CCL13) follow a similar pattern, with a higher LR-score in immune and monocytic lineage-infiltrated SDCs. CCR5 interactions were reported to potentiate the recruitment of CCR5-expressing TAMs, including immunosuppressive characteristics. Together with enhanced DNA repair, CCR5 signaling may induce a pro-inflammatory and pro-metastatic immune phenotype, thereby conveying resistance to DNA-damaging agents 46. CCR5 inhibitors (maraviroc and leronlimab) were approved by the FDA. Clinical trials are currently underway for the use of these inhibitors in combination with either immune-checkpoint inhibitors or chemotherapy for the treatment of colorectal cancer (NCT01736813, NCT03274804, NCT03631407) and triple-negative breast cancer (TNBC) (NCT03838367). OX40L (TNFSF4) and receptor (TNFRSF4) expression were correlated with fibroblasts, T cells and the monocytic lineage.

## Discussion

A functional genomic study was conducted where human SDCs were compared to adjacent non-tumoral tissue by proteomics and transcriptomics (**Figure 1**). This analysis revealed deregulation of numerous genes involved in ECM remodeling and cancer-associated fibroblast (CAF) proliferation. Additionally, these genes are known not only for facilitating progression towards aggressiveness, EMT, and angiogenesis, but also for inducing the simultaneous expression of inflammatory and immunosuppressive pathways. Notch and TGF-β signaling were activated and are likely to contribute to the SDC TME 21,22,24. In mice, overexpression of TGF-β in normal salivary glands indeed causes ECM remodeling and the replacement of normal glandular parenchyma with interstitial fibrous tissue 35.

Given the strong ECM remodeling signature determined by transcriptomics and proteomics, SDC DSR was assessed by measuring α-SMA abundance. As expected, a large proportion of SDC with DSR grades ≥ 2 (86%) was defined. Interestingly, this was independent of the immune-infiltrate phenotype (**Figure S7**).

The combination of immune cell gene signatures and T cell IHC quantitation and localization (CD3+ and CD8+) defined two groups of SDC: immune-poor (36% of SDCs) *versus* immune-infiltrated (64%) (**Figure 2**). An increase in TAM concentration was observed in the infiltrated group, where more than 50% of the macrophages were M2. Depending on the localization of the T cells, *i.e.*, present in the TC (46%) or restricted to the IM (18%), two sub-groups for immune-infiltrated SDC existed. When T cells were present in the TC, they were concentrated and maintained at the periphery of tumor nests, a structure that has been previously observed and could be due to TAMs and stromal cells 27,28. Based on T cells, the immune-poor group resembles immune-deserted tumors 26,37. The characterization of SDC immune-infiltrate phenotypes establishes a first concept for patient segregation with respect to immunotherapy. Immune-poor tumors are obviously less likely to benefit from such treatments.

We developed an algorithm to map TME intercellular interactions that could be disrupted. This algorithm identified 179 confident predictions of active LR pairs, which covered immune and non-immune functions (**Figure 4**). A total of 72 LR pairs were identified that correlated with SDC immune phenotypes and depicted a complex network of mixed pro- and anti-inflammatory interactions (**Figure S8**). Three immune checkpoints with existing inhibitory molecules were selected for validation by multiplexed IF. These were the PD-1/PD-L1 interaction, that has already been evaluated in SDCs 16 and tested in advanced salivary gland carcinomas 14, as well as the CTLA-4/DC86 and TIM-3/galectin-9 interactions. These last two interactions have never been previously evaluated in SDC. The three interactions were found to be significantly correlated with the immune phenotype (**Figure S11**), experimentally confirmed by ligand-receptor co-localization (**Figure 4E**). It was also shown that each ligand was produced by TAMs (**Figure 4F**). Thus, further evidence suggesting that TAMs play an important role in SDC immunosuppression. For the TIM-3/galectin-9 interaction, NK cells were additionally identified as a source of ligand that is consistent with interferon-γ expression (**Figure 1**). In fact, the data even suggested a complex self- and cross-inhibition of macrophages and NK cells (**Figure S10**) that, upon disruption, could unleash concomitant antitumor responses. Indeed, monoclonal antibodies against galectin-9 reduced TAMs towards an M1 phenotype *in vitro* 38, whereas TIM-3 blockade increased intratumoral NK cell cytotoxicity in mice with MHC class I-deficient tumors 34 (a characteristic shared by SDCs 16).

Our results have obvious clinical implications. The definition of two major immune phenotypes based on CD8+ T cells suggests that SDC patients could be stratified after initial surgery for potential immunotherapy, similar to practice for many other tumors 26,37,39. Immunotherapies could be offered to immune-infiltrated patients that would target one of the immune checkpoints validated here. Limited data on advanced salivary gland carcinoma and SDC response to anti-PD-1 monotherapy indicate a low response rate (11% with pembrolizumab, KEYNOTE-028 trial 14). This modest outcome contrasts results with other tumors such as melanoma, where anti-PD-1 monotherapies were much more successful (see **Figure S12** for a comparison of PD-1/PD-L1 LR-scores in melanoma). In SDC, combined approaches might be considered, *e.g.*, anti-PD-1/PD-L1 and anti-CLTA-4 therapy. Moreover, given the role of NK cells, strategies exploiting the innate immune system should also be considered 34,41.

The dense stroma of SDC could also be addressed. For instance, in a murine pancreatic ductal adenocarcinoma (PDAC) model, depletion of FAP-expressing CAFs is synergized with anti-PD-L1 immunotherapy 42. In metastatic urothelial cancers, efficacy of anti-PD-L1 antibodies was improved by reducing TGF-β signaling in stromal cells and improving the penetration of T cells in the tumor 21. In PDAC, it has also been proposed that focal adhesion kinase inhibitors can be used to improve checkpoint immunotherapy efficacy 43. Stroma targeting could be even more substantial for SDC devoid of immune infiltrate. Targeting hyaluronic acid (HA) with PEGPH20, a pegylated recombinant human hyaluronidase, has resulted in better delivery of small molecule therapy in PDAC 44. Phase 2 and phase 3 trials of PEGPH20 combined with chemotherapy are underway in metastatic PDAC.

This study is the first attempt to profile the SDC microenvironment and combine the information gained from functional genomics via comprehensive transcriptomic, proteomic, and digital imaging analyses on both patient tumoral and non-tumoral tissue. We put forward a large repertoire of cellular interactions that could be disrupted, thus complementing existing research by others on the characterization of the actionable mutations of this tumor. Based on the immune infiltrate, two groups of SDC were identified, providing a rationale for patient enrolment in clinical trials. The importance of macrophages, NK cells and potentially Tregs, was also shown and should be taken into consideration to better define patient groups. Moreover, upregulated pathways were discovered that could be targeted, *i.e.*, Notch and TGF-β. The clear trends between M2 macrophage or α-SMA abundance and RFS could be developed into tools to better manage patient treatment following tumor resection. In addition, our work unravels novel treatment options for SDC devoid of immune infiltrate by targeting the stroma.

## Supplementary Material

Supplementary materials and methods, figures, and tables.

## Figures and Tables

**Table 1:** Clinical information of 24 SDC patients (cohorts 1 and 2).

Clinical Feature	n (%) or mean (range)
Male	16 (67%)
Age at diagnosis	64 (33 - 87)
Cardiovascular risk	11 (40%)
**Primary tumor site**	
Parotid	22 (92%)
Submandibular gland	1 (4%)
Submaxillary gland	1 (4%)
**T classification**	
T2	8 (33%)
T3	4 (17%)
T4 (including 4a and 4b)	12 (50%)
**N classification**	
N0	7 (29%)
N1	2 (8%)
N2	1 (4%)
N2b	11 (46%)
Unknown	3 (13%)
**M classification**	
M0	9 (38%)
M1	3 (12%)
Unknown	12 (50%)
**Initial therapy**	
Surgery+RT	14 (58%)
Surgery+RT+CT	8 (34%)
Surgery	2 (8%)
**Disease Recurrence**	
No	6 (25%)
Local and regional	1 (4%)
Local and distant	3 (13%)
Regional	1 (4%)
Distant	8 (33%)
Deceased before recurrence	2 (8%)
Recent cases (unevaluable)	3 (13%)
**Second line therapy**	
Radiotherapy	1 (8%)
Chemotherapy	5 (38%)
Surgery	4 (31%)
Immunotherapy	1 (8%)
Unknown	2 (15%)

## Abbreviations

ADT: Androgen deprivation therapy
AR: Androgen receptor
CAF: Cancer-associated fibroblast
CNA: Copy number alteration
DDA: Data-dependent acquisition
DIA: Data-independent acquisition
ECM: Extracellular matrix
EMT: Epithelial to mesenchymal transition
FDR: False discovery rate
GO: Gene ontology
HA: Hyaluronic acid
IDC: Invasive ductal mammary carcinoma
IF: Immunofluorescence
IHC: Immunohistochemistry
IM: Invasive margin
LR: Ligand-receptor
PDAC: Pancreatic ductal adenocarcinoma
RFS: Recurrence- free survival
RT: Room temperature
SDC: Salivary duct carcinoma
TAM: Tumor-associated macrophages
TC: Tumor core
TCGA: The cancer genome atlas
TME: Tumor microenvironment
