# Supplementary material for: The molecular landscape and microenvironment of salivary duct carcinoma reveal new therapeutic opportunities
Source: Theranostics. 2020 Mar 15;10(10):4383–94. doi: 10.7150/thno.42986 (PMC7150470; doi:10.7150/thno.42986)
Supplement: Supplementary file 1 — Supplementary materials and methods, figures, and tables. [file thnov10p4383s1.pdf]

SUPPLEMENTARY MATERIAL

The molecular landscape and microenvironment of salivary duct carcinoma reveal new therapeutic opportunities

Melissa Alame[1,2,3,4], Emmanuel Cornillot[1,3,4,11], Valère Cacheux[2,5,11] , Guillaume Tosato[1,3,4], Marion Four[6], Laura De Oliveira[6], Stéphanie Gofflot[7], Philippe Delvenne[8], Evgenia Turtoi[1,4,9], Simon Cabello-Aguilar[1,4,9], Masahiko Nishiyama[10], Andrei Turtoi[1,4,9,12], Valérie Costes-Martineau[5,6,12], Jacques Colinge[1,4,5,12]

[1] Institut de Recherche en Cancérologie de Montpellier (IRCM), INSERM, Parc Euromédecine, 208 rue des Apothicaires, 34298 Montpellier, France
[2] Biological Hematology Department, CHU Montpellier, Hôpital Saint Eloi, 34275 Montpellier, France
[3] Université de Montpellier, Faculté de Pharmacie, 15 avenue Charles Flahault, 34093 Montpellier, France
[4] Institut Régional du Cancer Montpellier (ICM), Parc Euromédecine, 208 rue des Apothicaires, 34298 Montpellier, France
[5] Université de Montpellier, Faculté de Médecine, 2 rue école de Médecine, 34060 Montpellier, France
[6] Biopathology Department, CHU Montpellier, Hôpital Gui De Chauliac, 34000 Montpellier, France
[7] Biothèque, Université de Liège, 4000 Liège, Belgium
[8] Pathology Department, CHU Liège, Université de Liège, 4000 Liège, Belgium
[9] Université de Montpellier, 163 rue Auguste Broussonnet, 34090 Montpellier, France
[10] Department of Molecular Pharmacology and Oncology, Gunma University Graduate School of Medicine, Gunma, Japan
[11] Equal contribution
[12] Corresponding authors: andrei.turtoi@inserm.fr, v-costes_martineau@chu-montpellier.fr, jacques.colinge@inserm.fr

Running title: Molecular landscape and microenvironment of SDC

Keywords: salivary duct carcinoma, stroma, personalized medicine, immunotherapy, molecular pathways

Supplementary Materials and Methods

Patient consents

The French patients with SDC diagnosis were consented for tissue collection and research analysis under institutional reviewing board approval at the University Hospital of Montpellier (France). For the Belgian patients, the ethical committee of the University Hospital Liege has approved the use of human material in the current study. All samples were obtained from the institutional biobank of the University Hospital Liege, Belgium. According to Belgian law, patients obtained the information that the residual material could be used for research purpose and the consent is presumed as long as the patient does not oppose (opting-out), which was not the case for those patients.

Transcriptomics

Cohort 1 fresh frozen sample RNA was extracted using DNeasy Blood and Tissue Kit (Qiagen) and quantified by spectrophotometry using the Nanodrop 2000 (ThermoFisher Scientific). RNA quality and integrity was analyzed by 2100 Bioanalyzer (Agilent) and Fragment Analyzer. DNA libraries were prepared with the NEBNext Ultra II mRNA-Seq kit. Quantification of the library was obtained by real-time PCR. Sequencing and data processing methods are detailed in the main paper.

PolyA+ selection and rRNA depletion are the main approaches for RNA preparation in RNA-seq studies. We used polyA+ selection and the MSKCC cohort was prepared with RiboErase rRNA depletion. Among coding genes, histone genes are known to be difficult to quantify with polyA+ selection, whereas the other genes are quantified almost identically with the two approaches [1]. Searching for 50-fold or higher variation between cohort 1 and cohort MSKCC after upper quartile read count normalization yielded a list of 41 genes that was comprised of histones mostly:

Table S1. Most affected genes by differences in sample preparation, cohorts 1 *vs.* MKSCC.

AC009022.1	HIST1H2AI	HIST1H3C	HIST2H2AA4
AC087392.1	HIST1H2AJ	HIST1H3F	HIST2H2AB
AL138751.1	HIST1H2AL	HIST1H3I	HIST2H2AC
AL139333.1	HIST1H2BB	HIST1H3J	HIST2H3A
CTD-2116N17.1	HIST1H2BI	HIST1H4A	HIST2H3C
HIST1H1A	HIST1H2BL	HIST1H4B	HIST2H4B
HIST1H1B	HIST1H2BM	HIST1H4C	OR2D2
HIST1H1D	HIST1H2BO	HIST1H4D	OR6A2
HIST1H1E	HIST1H3A	HIST1H4F	TAS2R50
HIST1H2AB	HIST1H3B	HIST1H4L	UBQLN3
HIST1H2AH			

We decided to remove these 41 genes from the study. The final read count matrix combining cohorts 1 and MSKCC was filtered by only keeping the genes expressed with > 5 reads in > 5 samples (16,680 genes). Subsequently, the matrix was normalized by total read counts. We observed no significant batch effect between cohorts (**Figure S1**). It was also the case in all our subsequent analyses.

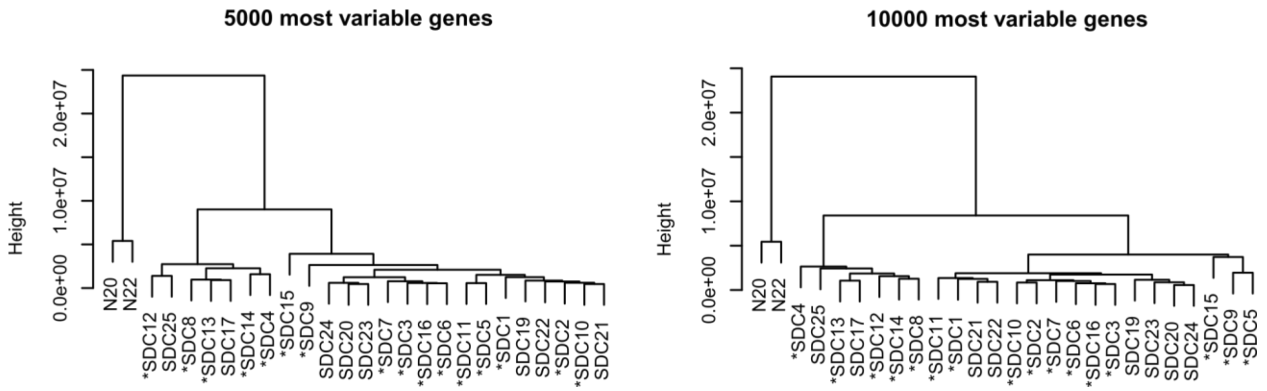

Figure S1. Absence of batch effect. We considered genes with minimal expression at least, imposing an average of 10 reads over all the samples (after data normalization), which left us with 16,129 genes. Then, we selected either the 10,000 or the 5,000 most variable genes based on their coefficient of variation and computed a dendrogram. Both dendrograms showed perfect separation of the normal samples from SDCs. They also mixed Dalin et al. samples (marked with an asterisk) and ours in the different parts of the dendrograms, showing the absence of any notable batch effect. The two dendrograms also clustered samples in a comparable fashion, which was obviously expected.

Proteomic Analysis of FFPE Samples

Twenty FFPE tissue sections of five-micrometer thickness were deparaffinized with 1ml of xylen at 60°C for 10 min. Following this, the samples were centrifuged at 20.000g, room temperature (RT) for 5 min and the supernatant was removed. The xylen treatment was reapplied for a total of 3 times. Next, 1ml of ethanol was added and the samples were vortexed and centrifuged at 20.000g, RT for 5 min. The supernatant was discarded and the ethanol wash was re-applied to the pellet for a total of 4 times. The samples were then dried using Speed Vacuum and suspended in 500µl of citrate buffer (pH 6) with 1% SDS. Following a sonication step, the samples were incubated for 30min at 95°C under vigorous shaking. Next, the samples were allowed to cool down at RT for 20-30min and the pH was re-adjusted to 8.5 with 100mM NaOH solution. The samples were centrifuged (20.000g, RT for 5 min) and the supernatant was transferred to a new tube. The protein content of the resulting solution was determined using BCA Protein Quantification Kit (Thermo Fisher, Waltham, MA, USA; cat. no.: 23225). Hundred microgram of protein extract was transferred in a fresh tube and subjected to reduction using 20mM DTT for 30min at 60°C. Following this, the protein samples were alkylated using 50mM 2-chloroacetamide for 30min at RT, in the absence of light and under shaking. The proteins were then precipitated using 2D Clean-Up kit according to the manufacturer's instructions (GE Healthcare, Chicago, IL, USA; cat. no. 80648451). The protein pellets were then suspended in 50µl of ammonium bicarbonate 100mM

/calcium chloride 1mM buffer (pH 8). To this suspension, 0.01% of Protease Max surfactant was added along with 1μg of trypsin. The samples were digested overnight (ON) at 37°C. Following digestion, 10% of each sample was transferred in a new tube where all the samples were mixed in a library. The remainder of the sample was Speed Vac to dryness. The library sample was further subjected to peptide fractionation using High pH Reversed-Phase Peptide Fractionation Kit according to the manufacturer's instructions (Thermo Fisher; cat. no.: 84868). From the library sample, 8 individual peptide fractions were derived, which were then Speed Vac to dryness. All the samples, including the library samples, were dissolved in 0.1% TFA and were subjected to salt removal using ZipTip according to the manufacturer's instructions (Merck, Darmstadt, Germany; cat. no.: C5737).

The peptide samples were analyzed using a 1D-nano-HPLC system (Sciex, Framingham, MA, USA), which was connected on-line with an electrospray Q-TOF mass spectrometer 6600 (Sciex). A total of 1 µg of sample was injected on the C18 analytical column (Acclaim® 75 µm x 150 mm, p/n: 162224; Dionex, California, USA) with a gradient of 0–40% phase B (90 % acetonitrile, 9.9 % water and 0.1 % formic acid) for 100 min at the flow rate of 0.3 µl/min. Two acquisition modes were used, data-dependent (DDA) for the measurement of the library and data-independent (DIA or SWATH) for the samples. In the DDA mode, mass spectral data were acquired over a mass range from 400 to 1600 m/z. One full MS scan was automatically followed by up to 30 MS/MS scans of the most intensive peptides found in this mass range (bearing +2 or +3 charges). The acquired data for each fraction of the library sample were merged and used for MS/MS database search with Protein Pilot software (Sciex). For the SWATH acquisitions, the DDA method was adapted using the automated method generator embedded in the Analyst software (Sciex). Protein identification and quantification were conducted using Peak View software and the previously generated protein library.

Immunohistochemistry & Immunofluorescence

Five-micrometer thick paraffin sections were deparaffinized in xylene, rehydrated in a series of graded methanol dilutions (100% - 95% - 70% - 50%) and washed in phosphate saline buffer (PBS) with 0.25% Triton X-100 (VWR Chemicals, Randor, PA, USA; cat. no.: 28817.295). The endogenous peroxidase activity was blocked with 10% hydrogen peroxide in methanol (Sigma Aldrich, St. Louis, MI, USA; cat. no.: 216763) for 30 min. Antigen retrieval was conducted using AR6 buffer (Perkin Elmer, Waltham, MA, USA; cat. no.: AR600250) for 10 min in a pressure cooker. The sections were blocked for 30 min in protein block serum-free solution (Agilent-Dako, Santa Clara, CA, USA; cat. no.: X0909) and incubated with the primary antibody at RT for 2h. The list of antibodies used in the present work is outlined in **Table S2** below. Following this, the slides were washed three times in PBS for 5 min and then incubated for 30 min at RT with secondary antibody Histofine MAX PO Multi (Nichirei, Tokyo, Japan; cat. no. 414152F) for mouse and rabbit antibodies and Histofine MAX PO G (Nichirei Bio, cat. no. 414162F) for antibodies of goat origin. Subsequently, the sections were washed three times for 5 min in PBS and then stained with 3,3'-diaminobenzidine (DAB). The latter solution was made by adding 10μL of DAB Chromogen to 1 mL of DAB Substrate Buffer (Agilent-Dako, cat. no.: GV800). The slides

were counter-stained in hematoxylin (Sigma Aldrich, cat. no.: MHS32) and mounted with Eukitt (Orsatech GmbH, Bobingen, Germany).

For immunofluorescence, tissue sections were prepared as described above with exception of primary antibody incubation that was conducted at 4°C and over night and the staining that has been performed using Opal system (Perkin Elmer, cat. no.: NEL810001KT). Following the primary antibody incubation, the slides were incubated with the corresponding secondary antibody as described above. The slides were then incubated with 100μL staining solution prepared from 2μL Opal dye and 98μL Amplifying Buffer. Following 10 min incubation, the slides were washed three times for 5 min in PBS and then subjected to microwave-assisted antibody removal. Slides were immersed in AR6 buffer and were treated in the microwave for 15 min, maintaining the heat close to the boiling point. After cooling and a wash in PBS buffer for 5 min, the tissues were re-blocked with for 30 min in protein block serum-free solution at RT. Tissues were then incubated with the next primary antibody and the staining procedure was repeated as described above using the following Opal dyes: 520, 570, 620 and 690. Finally, slides were mounted using VECTASHIELD® Antifade Mounting Medium with DAPI (Vector, Burlingame, USA).

ifLR-score calculation and usage

The determination of the percentage of receptor-expressing cells that are surrounded by sufficient ligand fluorescence relies on the computation of an immunofluorescence ligand-receptor score (ifLR-score) and the definition of a threshold above which the interaction is considered positive. Taking the PD-1/PD-L1 interaction as an example, we first determined the average diameters of PD-1+ and PD-L1+ cells independently (**Figure S2A**). This allowed us to define a crown-shaped area around each PD-1+ cell. The receptor abundance R is estimated by the average PD-1 fluorescence inside the inner disc that is centered on the PD-1+ cell and has the corresponding diameter. The ligand abundance L is estimated in the crown that has a width equal to half the diameter of a PD-L1+ cell. L represents the number of ligands close enough to the PD-1+ cell to engage inhibition. Empirically, we define

$$\text{ifLR-score} = L^{1/3}R^{1/2}/(M + L^{1/3}R^{1/2}),$$

where L and R are as above, M is the average of the average intensity over the whole ligand image and the average intensity over the whole receptor image (each label results in a separate gray-scale image). The fractional powers account for the ligand and the receptor to reside in a 3-, respectively 2-, dimensional space. M represents the background signal intensity and its role is to regularize the ifLR-score to obtain values between 0 and 1. Analysis of the PD-1/PD-L1 interaction in 3 SDC allowed us to plot ifLR-score value distribution (**Figure S2B**). It is bimodal (or even trimodal for SDC22), with a fist mode corresponding to random signals (low values) followed by a rightmost mode corresponding to overlapping ligand and receptor fluorescence. We empirically set a conservative threshold at 0.4. That is, each PD-1+ cell with ifLR-score > 0.4 is considered in positive interaction with adjacent PD-L1+ cell(s), otherwise the interaction is deemed negative.

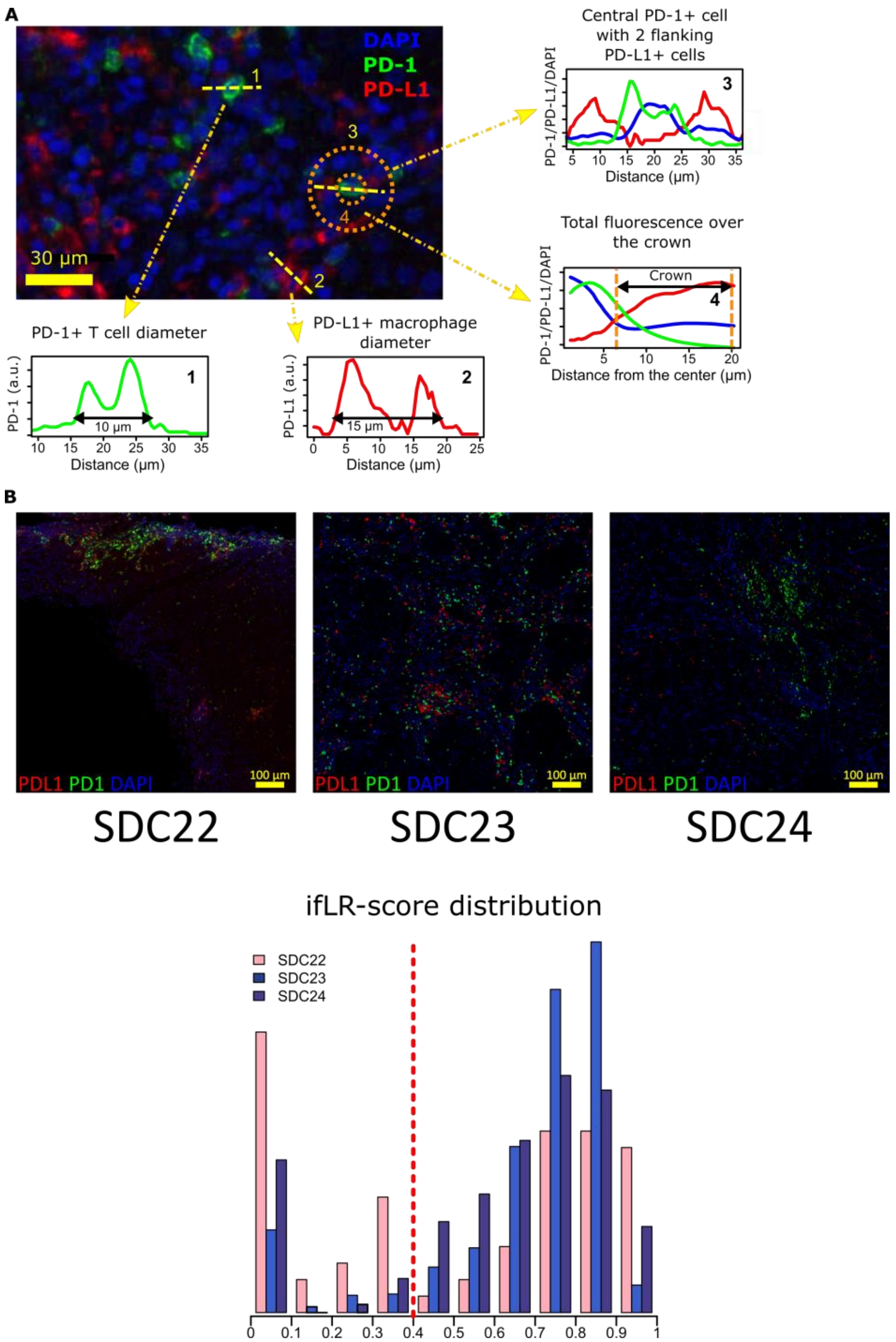

Figure S2. Principle of detecting ligand-positive and receptor-positive cell interactions exemplified by PD-1/PD-L1. (A) Average PD-1+ cell diameter was estimated from 20 cells along a crossing axis (1). We note the increased green signal at the membrane and its decrease at the center of the cell (nucleus). Same operation for PD-L1+ cells (read, 2). Over a PD-1+ cell with flanking, adjacent PD-L1+ cells, we note the coherent signals with DAPI in blue (3). To compute the ifLR-score, we use the green average signal inside the inner circle of the crown (PD-1+ cell diameter) and the red average signal in the crown that has a width equal to half a PD-L1+ cell diameter. (B) SDC IF images and ifLR-score distributions with the threshold as vertical dashed red line.

LR-score (transcriptomics)

We also defined a ligand-receptor score meant to assess co-expression in transcriptomics as a proxy for potential true interaction in the sample. The empirical formula is similar to the above:

$$\text{LR-score} = l^{1/3}r^{1/2}/(\mu + l^{1/3}r^{1/2}),$$

where l is the ligand read count in $\log_{10}$ (ligand transcript expression), r the receptor read count in $\log_{10}$, and μ the average $\log_{10}$ read count over all the genes and all the SDC transcriptomes. See above for the fractional powers and μ roles.

Additional Figures and Tables

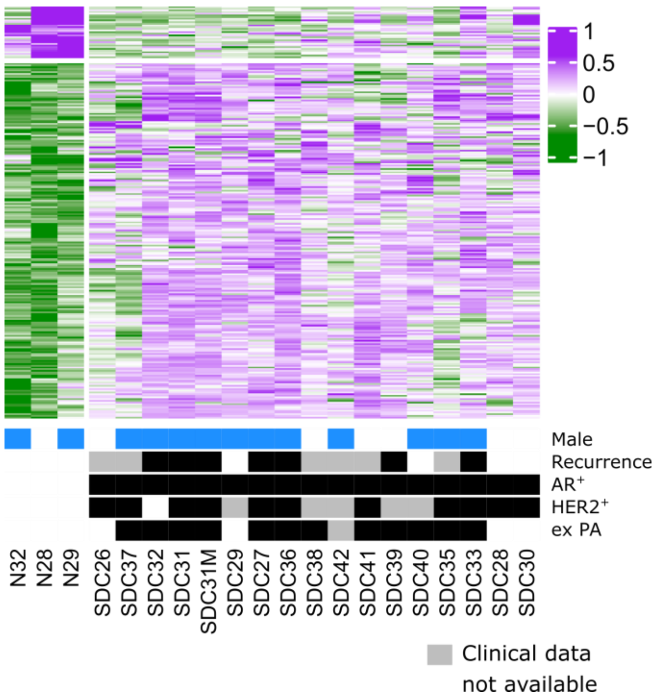

Figure S3. Differentially expressed proteins. (Missing recurrence data are due to limited follow-up time for recently enrolled patients).

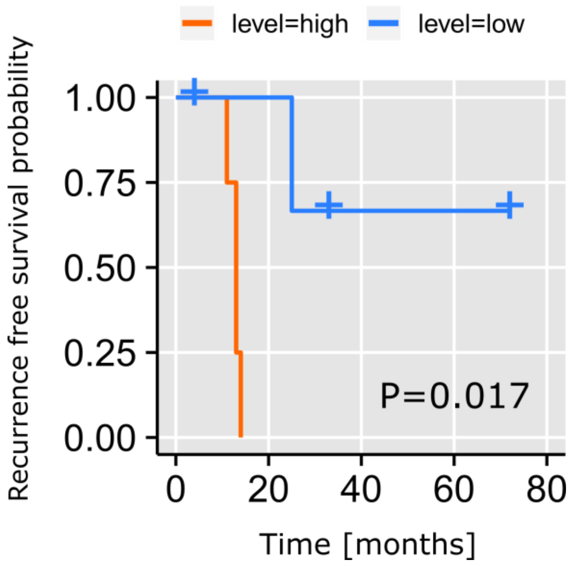

Figure S4. Recurrence-free survival of our cohort 1 with respect to *IFNG* expression level (Kaplan-Meier curves, log-rank test, n=8, high=above median, low=below median).

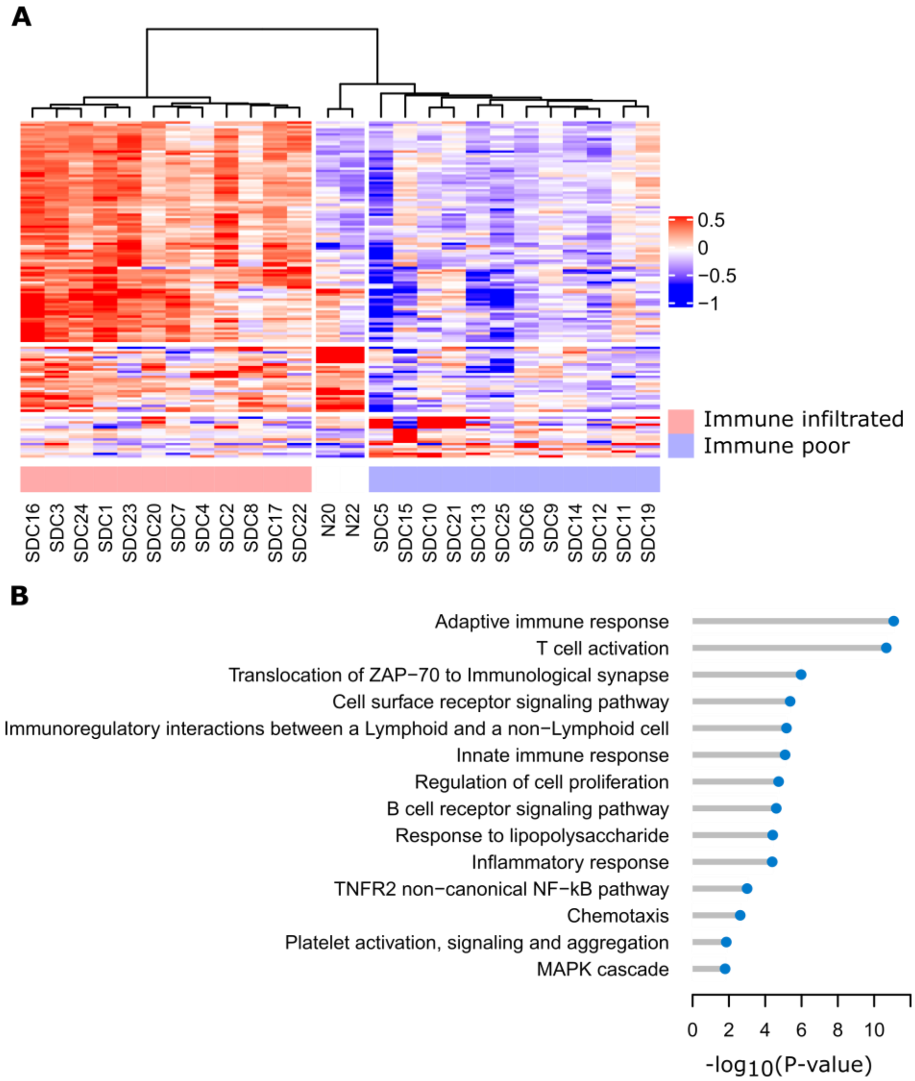

Figure S5. Differentially expressed genes between immune infiltrated and poor SDC. (A) The comparison selected 135 significantly regulated genes (FDR<0.01, log_2-FC>2 in absolute value, average read count>20), which segregate the two sample clusters perfectly (plus normal samples for reference). (B) Main GOBP terms and Reactome pathways significantly enriched (hypergeometric test, FDR<0.05, minimum 5 regulated genes in the GO term or the Reactome pathway).

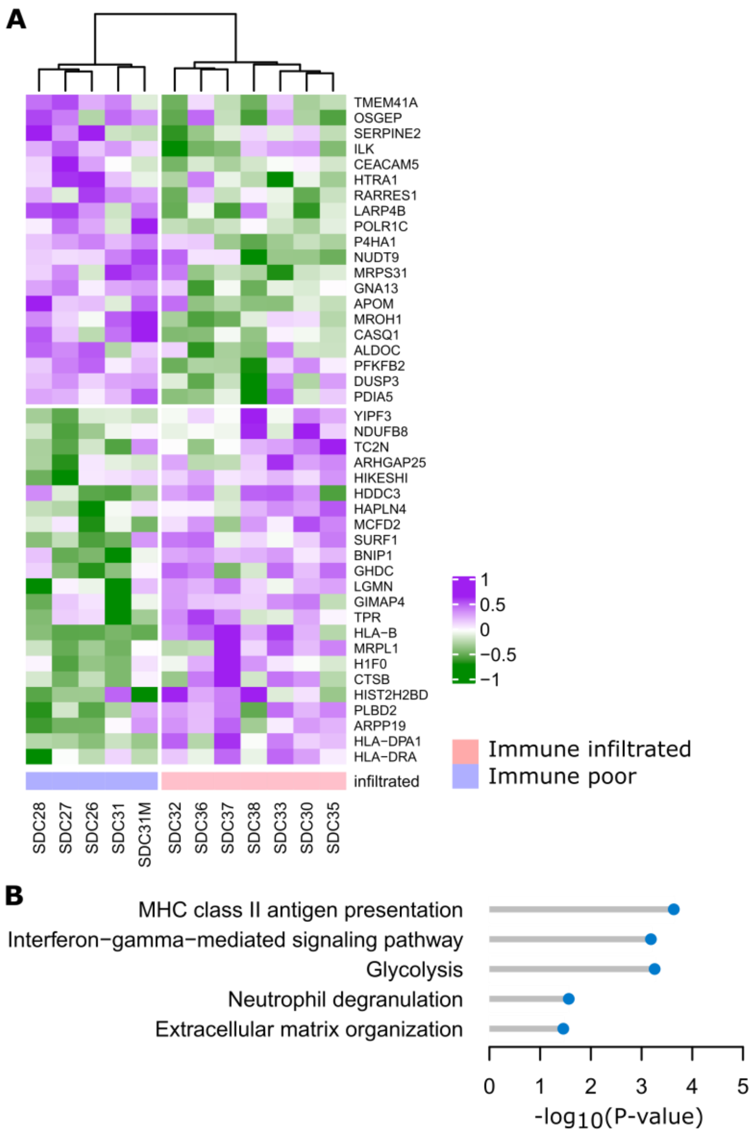

Figure S6. Differentially expressed proteins between immune infiltrated and poor SDC. (A) The comparison selected 43 significantly regulated genes (P<0.05, FC>1.5, average MS signal >3), which segregate the two sample clusters perfectly (plus normal samples for reference). Proteomics data were available for 12/14 cohort 2 samples. (B) Main GOBP terms and Reactome pathways significantly enriched (hypergeometric test, FDR<0.05, minimum 3 regulated proteins in the GO term or the Reactome pathway).

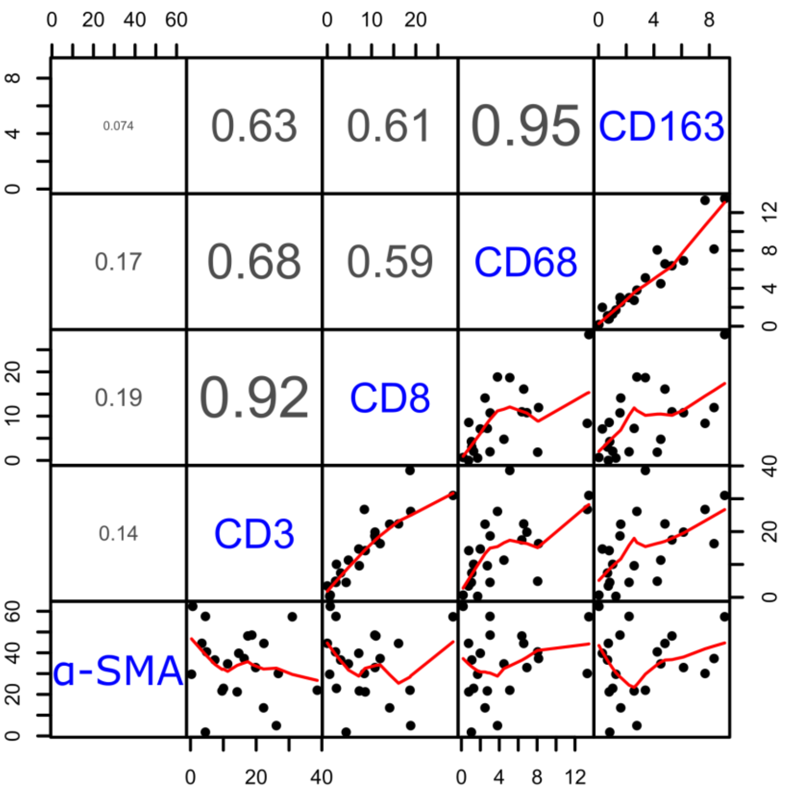

Figure S7. Spearman correlations between CD3, CD8, CD68, CD163, and α-SMA levels. We note the quasi-absence of correlation with α-SMA indicating an immune infiltrate that does not depend on the desmoplastic stromal reaction level in SDCs (n=22).

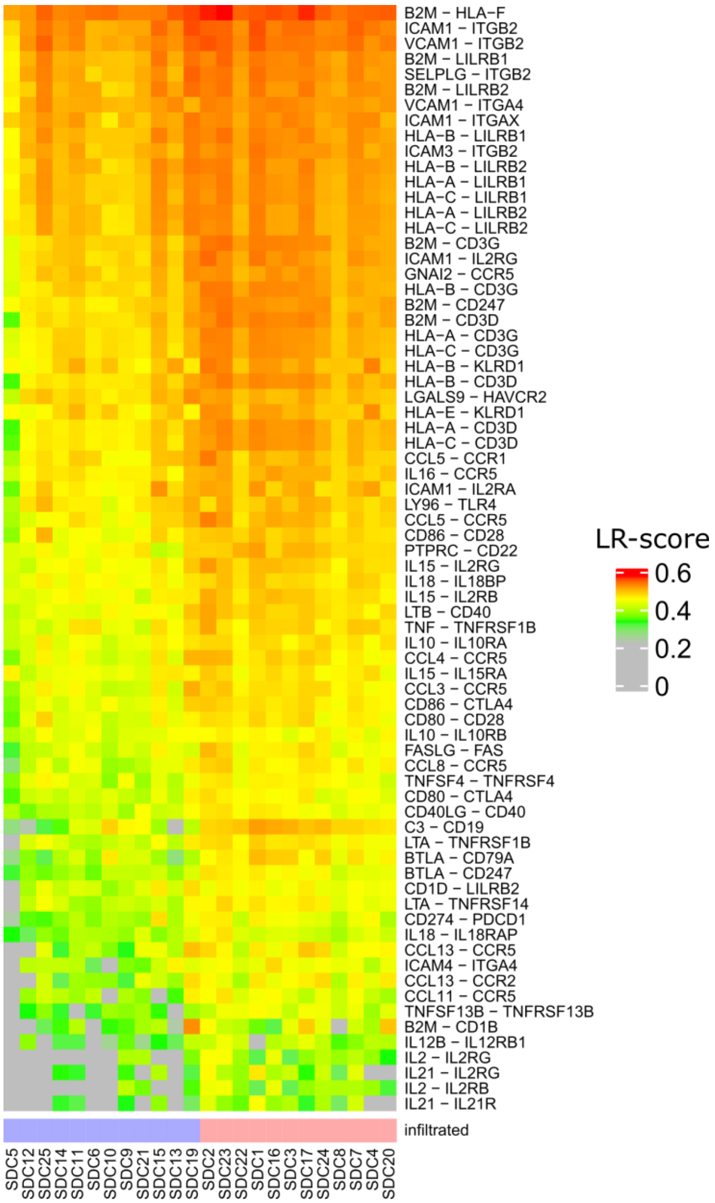

Figure S8. Confidence LR pairs whose transcriptomic LR-score are correlated with immune infiltrate levels of SDC (Spearman r>0.6, immune infiltrate level defined as the average MCP-counter T cells, B cells and CD8+ cells signatures).

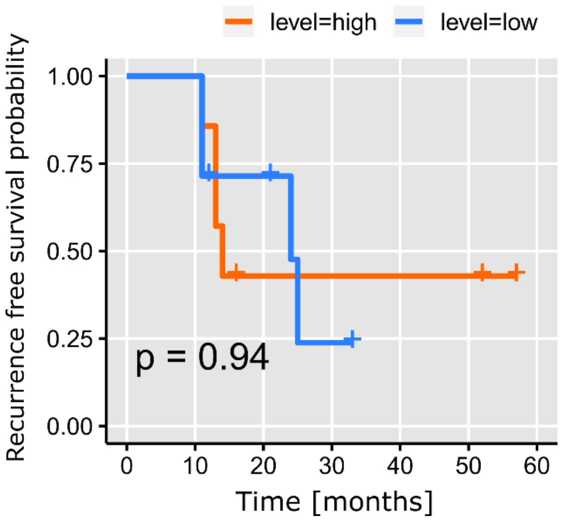

Figure S9. Recurrence free survival among the immune infiltrated SDC with respect to the percentage of CD8+ positive cells. We note no significant association (Kaplan-Meier curves, log-rank test, n=14, high=above median, low=below median).

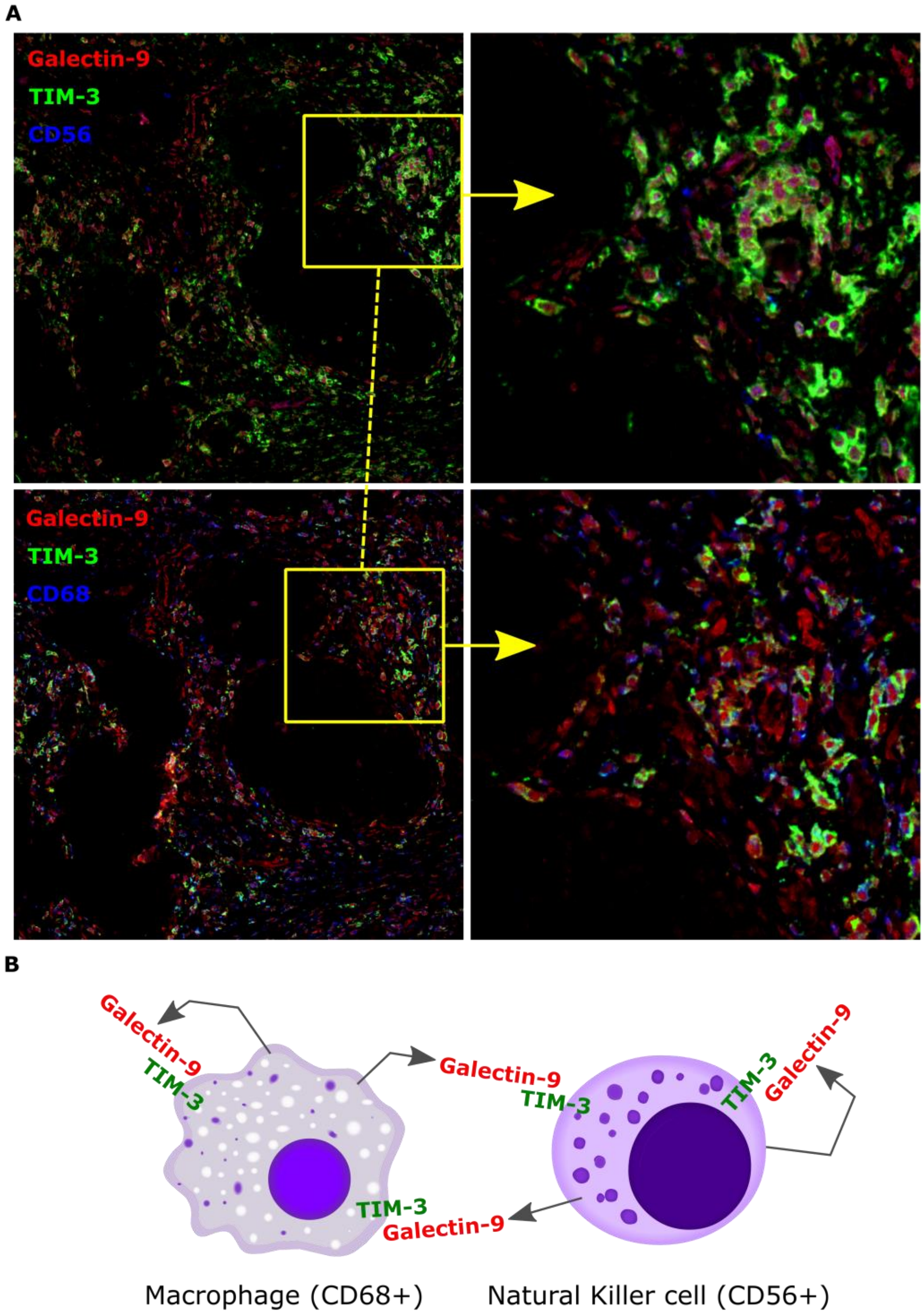

Figure S10. Co-localization patterns of CD56, CD68, Galectin-9, and TIM-3 fluorescence. (A) In adjacent FFPE block slides, we selected corresponding areas (yellow squares, left side). In both cases, we see individual cells positives for the markers (right side, CD68, Galectin-9, TIM-3 or CD56, Galectin-9, TIM-3) indicative of autocrine inhibition. Since the slides were adjacent and we selected the same areas, it additionally indicates simultaneous paracrine inhibition between macrophages and NK cells. (B) Schema of dual auto- and paracrine inhibition. (Cell cartoon source: commons.wikimedia.org).

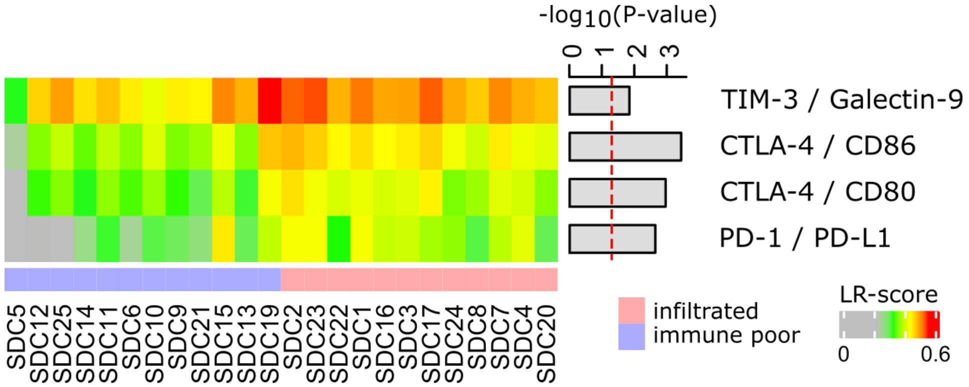

Figure S11. Correlation of the LR-scores of the immune checkpoints involving PD-1, CTLA-4, and TIM-3. P-values (Wilcoxon) infiltrated *versus* immune poor LR-scores are featured (5% significance = red dashed line). Immune poor SDC are not devoid of ligand and receptor transcripts since the classification is based on CD8+ T cells, but other immune cells express those immune checkpoints and their ligands, e.g., TAMs and NK cells (**Figure 4**).

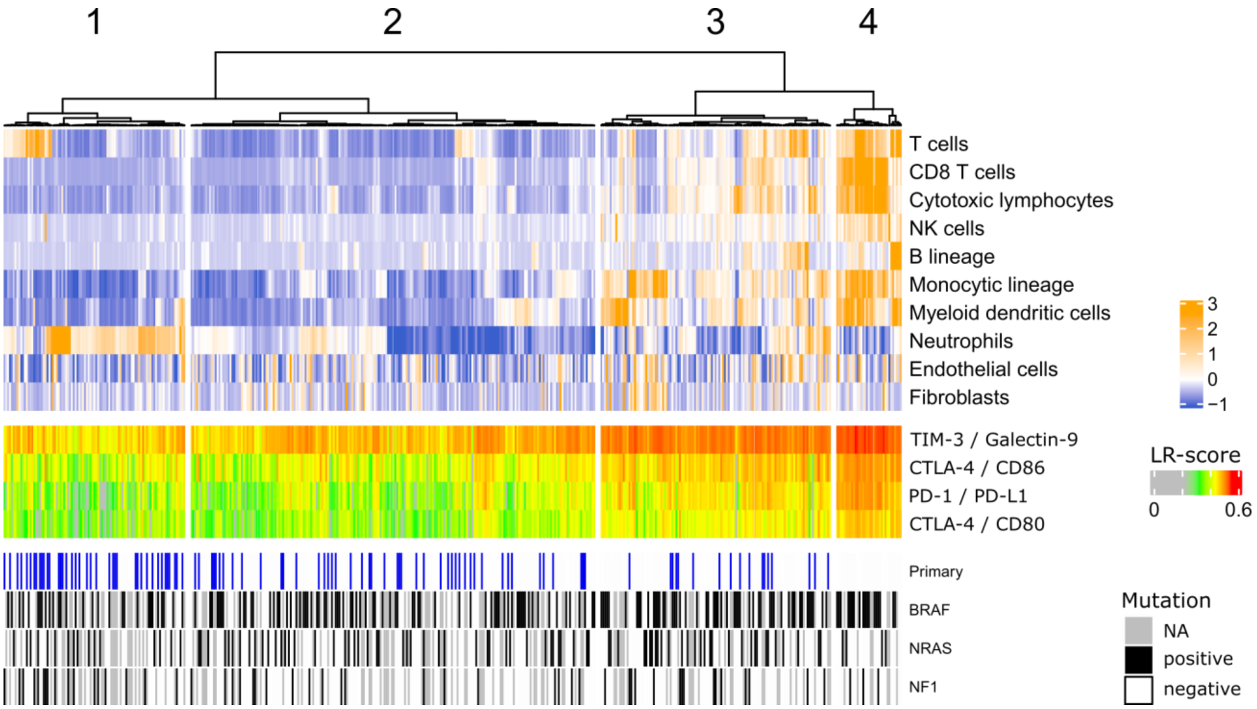

Figure S12. In melanoma, monotherapies with pembrolizumab or nivolumab, two anti-PD-1 agents, resulted in 26-59% responses (overall survival at 2 years), *e.g.*, CheckMate-037 & -066 and KEYNOTE-001 & -002 clinical trials. To compare with SDC, we retrieved 471 tumors from TCGA SKCM cohort and applied the same data analysis we did with SDCs. MCP-counter identified a large cluster (union of clusters 3 & 4) with general immune infiltrate, including a subcluster (4) with strong CD8+ T cell infiltrate. Cluster 1, enriched in primary tumors, contains limited immune infiltrate comprised of T cells and neutrophils. The four LR pairs discussed further in SDC clearly correlate with immune cells, especially the presence of CD8+ T cells. PD-1/PD-L1 LR-scores in CD8+ T cell-rich tumors (cluster 4) are stronger than their counterparts in SDC (**Figure S11**). No correlation with common melanoma mutations. No correlation with neutrophils.

Table S2. References and dilutions of primary antibodies used for IHC and IF.

Target	Manufacturer	Cat. No.	Dilution IHC	Dilution IF
CD3	Dako	GA503	undiluted	1/3
CD8	Roche	5937248001	undiluted	N/A
CD68	Dako	M0814	1/5000	1/1500
PD-L1	Dako	M3653	undiluted	1/3
CD163	Roche	7604437	undiluted	1/3
α-SMA	Dako	M0851	1/500	N/A
TIM3	Cell Signalling Technology	45208	N/A	1/150
PD-1	Cell Signalling Technology	86163	N/A	1/300
Galectin-9	Cell Signalling Technology	54330	N/A	1/2500
Pan-Cytokeratin	Dako	GA053	N/A	1/3
CTLA-4	Abcam	ab227709	undiluted	1/50

Table S3. Interactions added to Reactome binary interactions as retrieved from PathwayCommons. Information taken from UniprotKB/Swissprot.

Receptor	Interactor	Receptor	Interactor	Receptor	Interactor
HAVCR2	LCK	TNFRSF4	TRAF2	TNFRSF18	TRAF2
HAVCR2	PLCG	TNFRSF4	TRAF3	TNFRSF18	TRAF3
HAVCR2	VAV1	TNFRSF4	TRAF5	TNFRSF18	SIVA1
HAVCR2	AKT1	TNFRSF8	TRAF1	TNFRSF25	TNFRSF1
HAVCR2	AKT2	TNFRSF8	TRAF2	TNFRSF25	TRADD
HAVCR2	LCP2	TNFRSF8	TRAF3	TNFRSF25	BAG4
HAVCR2	ZAP70	TNFRSF8	TRAF5		
HAVCR2	SYK	TNFRSF9	TRAF1		
HAVCR2	PIK3R1	TNFRSF9	TRAF2		
HAVCR2	FYN	TNFRSF9	TRAF3		
HAVCR2	SH3BP2	TNFRSF9	LRR1		
HAVCR2	SH2D2A	TNFRSF18	TRAF1		

Table S4. Enriched Reactome pathways and GOBP terms in regulated genes.

term	description	intersect.size	n.pw	pval	qval
R-HSA-68877	Mitotic Prometaphase	59	193	0.00E+00	0.00E+00
R-HSA-69620	Cell Cycle Checkpoints	78	273	0.00E+00	0.00E+00
GO:0051301	cell division	85	355	0.00E+00	0.00E+00
R-HSA-194315	Signaling by Rho GTPases	96	409	0.00E+00	0.00E+00
R-HSA-69278	Cell Cycle, Mitotic	128	493	0.00E+00	0.00E+00
R-HSA-1640170	Cell Cycle	144	598	0.00E+00	0.00E+00
R-HSA-68886	M Phase	84	352	1.11E-16	5.36E-14
R-HSA-2500257	Resolution of Sister Chromatid Cohesion	44	122	2.22E-16	9.39E-14
R-HSA-195258	RHO GTPase Effectors	71	278	4.44E-16	1.67E-13
R-HSA-5663220	RHO GTPases Activate Formins	46	135	6.66E-16	2.25E-13
R-HSA-141424	Amplification of signal from the kinetochores	37	93	1.55E-15	4.38E-13
R-HSA-141444	Amplification of signal from unattached kinetochores via a MAD2 inhibitory signal	37	93	1.55E-15	4.38E-13
GO:0007062	sister chromatid cohesion	39	108	1.24E-14	3.23E-12
R-HSA-69618	Mitotic Spindle Checkpoint	38	109	9.93E-14	2.40E-11
R-HSA-68882	Mitotic Anaphase	51	194	2.09E-12	4.72E-10
R-HSA-2555396	Mitotic Metaphase and Anaphase	51	195	2.59E-12	5.48E-10
R-HSA-2467813	Separation of Sister Chromatids	48	186	1.90E-11	3.79E-09
GO:0000281	mitotic cytokinesis	24	59	8.41E-11	1.58E-08
GO:0007059	chromosome segregation	25	67	3.10E-10	5.52E-08
GO:0007019	microtubule depolymerization	11	14	9.73E-10	1.65E-07
R-HSA-176974	Unwinding of DNA	10	12	2.13E-09	3.43E-07
GO:0007052	mitotic spindle organization	18	40	2.72E-09	4.19E-07
GO:0006260	DNA replication	36	136	2.94E-09	4.32E-07
R-HSA-1474244	Extracellular matrix organization	60	300	4.19E-09	5.90E-07
R-HSA-1474290	Collagen formation	27	90	1.54E-08	2.08E-06
GO:0008283	cell proliferation	67	367	2.50E-08	3.25E-06
GO:0006270	DNA replication initiation	16	36	2.61E-08	3.26E-06
R-HSA-69190	DNA strand elongation	15	32	2.93E-08	3.54E-06
R-HSA-69481	G2/M Checkpoints	36	151	5.69E-08	6.64E-06
R-HSA-69306	DNA Replication	32	127	7.77E-08	8.76E-06
R-HSA-606279	Deposition of new CENPA-containing nucleosomes at the centromere	19	54	1.25E-07	1.28E-05
R-HSA-774815	Nucleosome assembly	19	54	1.25E-07	1.28E-05
GO:0000278	mitotic cell cycle	31	123	1.23E-07	1.28E-05
GO:0007018	microtubule-based movement	31	126	2.20E-07	2.19E-05
GO:0000070	mitotic sister chromatid segregation	14	32	2.49E-07	2.41E-05
R-HSA-73886	Chromosome Maintenance	25	90	2.73E-07	2.57E-05
R-HSA-176187	Activation of ATR in response to replication stress	15	37	3.12E-07	2.85E-05
R-HSA-68962	Activation of the pre-replicative complex	14	33	3.96E-07	3.53E-05
R-HSA-453279	Mitotic G1-G1/S phases	33	148	1.03E-06	8.96E-05

R-HSA-2022090	Assembly of collagen fibrils and other multimeric structures	19	61	1.08E-06	9.16E-05
R-HSA-1650814	Collagen biosynthesis and modifying enzymes	20	67	1.21E-06	9.98E-05
R-HSA-983189	Kinesins	19	62	1.43E-06	1.15E-04
GO:0007051	spindle organization	11	23	1.66E-06	1.31E-04
GO:0030199	collagen fibril organization	15	42	2.12E-06	1.63E-04
R-HSA-453274	Mitotic G2-G2/M phases	39	196	2.39E-06	1.80E-04
GO:0030574	collagen catabolic process	19	65	3.15E-06	2.32E-04
R-HSA-6791312	TP53 Regulates Transcription of Cell Cycle Genes	16	49	3.80E-06	2.74E-04
GO:0007080	mitotic metaphase plate congression	14	39	4.32E-06	3.05E-04
GO:0000086	G2/M transition of mitotic cell cycle	29	130	4.52E-06	3.06E-04
R-HSA-69206	G1/S Transition	29	130	4.52E-06	3.06E-04
R-HSA-1630316	Glycosaminoglycan metabolism	28	124	5.12E-06	3.40E-04
GO:0034080	CENP-A containing nucleosome assembly	12	30	5.69E-06	3.70E-04
R-HSA-69242	S Phase	33	160	6.28E-06	4.01E-04
R-HSA-69239	Synthesis of DNA	27	119	6.83E-06	4.28E-04
R-HSA-71387	Metabolism of carbohydrates	47	268	9.09E-06	5.59E-04
R-HSA-2243919	Crosslinking of collagen fibrils	9	18	9.58E-06	5.78E-04
GO:0010389	regulation of G2/M transition of mitotic cell cycle	21	82	9.83E-06	5.83E-04
R-HSA-69275	G2/M Transition	37	194	1.18E-05	6.90E-04
GO:0006281	DNA repair	39	211	1.49E-05	8.55E-04
R-HSA-8854518	AURKA Activation by TPX2	19	72	1.62E-05	9.15E-04
GO:0030198	extracellular matrix organization	37	197	1.69E-05	9.38E-04
R-HSA-68874	M/G1 Transition	21	85	1.79E-05	9.44E-04
R-HSA-69002	DNA Replication Pre-Initiation	21	85	1.79E-05	9.44E-04
R-HSA-216083	Integrin cell surface interactions	21	85	1.79E-05	9.44E-04
R-HSA-1793185	Chondroitin sulfate/dermatan sulfate metabolism	15	50	2.41E-05	1.25E-03
R-HSA-1566948	Elastic fibre formation	14	45	2.84E-05	1.45E-03
R-HSA-156711	Polo-like kinase mediated events	8	16	3.07E-05	1.55E-03
R-HSA-176417	Phosphorylation of Emi1	5	6	3.46E-05	1.68E-03
GO:0030206	chondroitin sulfate biosynthetic process	10	25	3.47E-05	1.68E-03
R-HSA-69273	Cyclin A/B1/B2 associated events during G2/M transition	10	25	3.47E-05	1.68E-03
R-HSA-212165	Epigenetic regulation of gene expression	25	116	3.73E-05	1.78E-03
GO:0043547	positive regulation of GTPase activity	53	332	3.89E-05	1.83E-03
R-HSA-1442490	Collagen degradation	17	64	4.08E-05	1.89E-03
R-HSA-3000171	Non-integrin membrane-ECM interactions	16	59	5.19E-05	2.37E-03
R-HSA-73894	DNA Repair	48	295	5.45E-05	2.46E-03
GO:0006367	transcription initiation from RNA polymerase II promoter	32	170	5.84E-05	2.60E-03
GO:0051056	regulation of small GTPase mediated signal transduction	28	141	6.27E-05	2.72E-03
R-HSA-194840	Rho GTPase cycle	28	141	6.27E-05	2.72E-03
R-HSA-5693538	Homology Directed Repair	25	120	6.75E-05	2.89E-03
GO:0007088	regulation of mitotic nuclear division	9	22	7.00E-05	2.92E-03

R-HSA-2565942	Regulation of PLK1 Activity at G2/M Transition	20	86	7.10E-05	2.93E-03
GO:0002040	sprouting angiogenesis	10	27	7.56E-05	3.03E-03
R-HSA-3000170	Syndecan interactions	10	27	7.56E-05	3.03E-03
R-HSA-4615885	SUMOylation of DNA replication proteins	13	43	7.62E-05	3.03E-03
GO:0000724	double-strand break repair via homologous recombination	19	80	7.89E-05	3.10E-03
GO:0051315	attachment of mitotic spindle microtubules to kinetochore	6	10	8.60E-05	3.34E-03
R-HSA-6804114	TP53 Regulates Transcription of Genes Involved in G2 Cell Cycle Arrest	8	18	8.84E-05	3.40E-03
R-HSA-3108232	SUMO E3 ligases SUMOylate target proteins	23	108	9.13E-05	3.47E-03
R-HSA-68867	Assembly of the pre-replicative complex	17	68	9.37E-05	3.52E-03
R-HSA-8948216	Collagen chain trimerization	13	44	9.92E-05	3.66E-03
GO:0006977	DNA damage response, signal transduction by p53 class mediator resulting in cell cycle arrest	16	62	9.94E-05	3.66E-03
GO:0007093	mitotic cell cycle checkpoint	10	28	1.08E-04	3.85E-03
R-HSA-539107	Activation of E2F1 target genes at G1/S	10	28	1.08E-04	3.85E-03
R-HSA-69205	G1/S-Specific Transcription	10	28	1.08E-04	3.85E-03
R-HSA-2980767	Activation of NIMA Kinases NEK9, NEK6, NEK7	5	7	1.12E-04	3.94E-03
GO:0097711	ciliary basal body-plasma membrane docking	21	96	1.21E-04	4.24E-03
R-HSA-983231	Factors involved in megakaryocyte development and platelet production	30	162	1.33E-04	4.60E-03
R-HSA-5620912	Anchoring of the basal body to the plasma membrane	21	97	1.42E-04	4.82E-03
R-HSA-68875	Mitotic Prophase	22	104	1.43E-04	4.82E-03
GO:0030203	glycosaminoglycan metabolic process	10	29	1.52E-04	5.03E-03
R-HSA-5693532	DNA Double-Strand Break Repair	28	148	1.51E-04	5.03E-03
R-HSA-2132295	MHC class II antigen presentation	25	126	1.54E-04	5.05E-03
GO:0019886	antigen processing and presentation of exogenous peptide antigen via MHC class II	21	98	1.65E-04	5.38E-03
R-HSA-68689	CDC6 association with the ORC:origin complex	6	11	1.75E-04	5.57E-03
R-HSA-2514853	Condensation of Prometaphase Chromosomes	6	11	1.75E-04	5.57E-03
GO:0021987	cerebral cortex development	15	59	1.95E-04	6.16E-03
GO:0071526	semaphorin-plexin signaling pathway	10	30	2.09E-04	6.54E-03
GO:0007094	mitotic spindle assembly checkpoint	8	20	2.15E-04	6.62E-03
R-HSA-2990846	SUMOylation	23	114	2.15E-04	6.62E-03
GO:0008360	regulation of cell shape	29	160	2.51E-04	7.64E-03
R-HSA-212300	PRC2 methylates histones and DNA	12	42	2.59E-04	7.83E-03
GO:0006268	DNA unwinding involved in DNA replication	5	8	2.76E-04	8.24E-03
GO:0007076	mitotic chromosome condensation	7	16	2.80E-04	8.24E-03
GO:0016571	histone methylation	7	16	2.80E-04	8.24E-03
R-HSA-5250913	Positive epigenetic regulation of rRNA expression	17	74	2.83E-04	8.25E-03
GO:0030334	regulation of cell migration	18	81	2.95E-04	8.52E-03
R-HSA-109582	Hemostasis	91	699	3.03E-04	8.67E-03
GO:0001578	microtubule bundle formation	9	26	3.14E-04	8.81E-03
GO:0051310	metaphase plate congression	6	12	3.22E-04	8.86E-03
GO:0032508	DNA duplex unwinding	12	43	3.30E-04	8.86E-03
R-HSA-177929	Signaling by EGFR	12	43	3.30E-04	8.86E-03

R-HSA-2299718	Condensation of Prophase Chromosomes	12	43	3.30E-04	8.86E-03
R-HSA-380259	Loss of Nlp from mitotic centrosomes	16	69	3.78E-04	9.99E-03
R-HSA-380284	Loss of proteins required for interphase microtubule organization from the centrosome	16	69	3.78E-04	9.99E-03
GO:0000082	G1/S transition of mitotic cell cycle	21	104	3.92E-04	1.03E-02
R-HSA-3000178	ECM proteoglycans	17	76	3.96E-04	1.03E-02
GO:0031100	animal organ regeneration	12	44	4.17E-04	1.08E-02
GO:0030335	positive regulation of cell migration	32	190	4.86E-04	1.24E-02
GO:0090307	mitotic spindle assembly	11	39	5.26E-04	1.32E-02
R-HSA-427389	ERCC6 (CSB) and EHMT2 (G9a) positively regulate rRNA expression	12	45	5.22E-04	1.32E-02
R-HSA-109581	Apoptosis	29	167	5.24E-04	1.32E-02
GO:2000573	positive regulation of DNA biosynthetic process	6	13	5.52E-04	1.34E-02
R-HSA-5140745	WNT5A-dependent internalization of FZD2, FZD5 and ROR2	6	13	5.52E-04	1.34E-02
R-HSA-73854	RNA Polymerase I Promoter Clearance	17	78	5.46E-04	1.34E-02
R-HSA-380320	Recruitment of NuMA to mitotic centrosomes	19	92	5.43E-04	1.34E-02
R-HSA-5693567	HDR through Homologous Recombination (HR) or Single Strand Annealing (SSA)	22	114	5.56E-04	1.34E-02
GO:0000463	maturation of LSU-rRNA from tricistronic rRNA transcript (SSU-rRNA, 5.8S rRNA, LSU-rRNA)	5	9	5.74E-04	1.37E-02
GO:0000733	DNA strand renaturation	5	9	5.74E-04	1.37E-02
GO:0051726	regulation of cell cycle	21	107	5.83E-04	1.37E-02
R-HSA-162582	Signal Transduction	288	2659	5.83E-04	1.37E-02
GO:0035987	endodermal cell differentiation	9	28	5.88E-04	1.37E-02
GO:0000083	regulation of transcription involved in G1/S transition of mitotic cell cycle	8	23	6.54E-04	1.50E-02
GO:1900182	positive regulation of protein localization to nucleus	8	23	6.54E-04	1.50E-02
R-HSA-5334118	DNA methylation	10	34	6.51E-04	1.50E-02
GO:0007221	positive regulation of transcription of Notch receptor target	7	18	6.62E-04	1.50E-02
R-HSA-5357801	Programmed Cell Death	29	170	7.06E-04	1.59E-02
R-HSA-380270	Recruitment of mitotic centrosome proteins and complexes	17	80	7.42E-04	1.62E-02
R-HSA-380287	Centrosome maturation	17	80	7.42E-04	1.62E-02
R-HSA-73864	RNA Polymerase I Transcription	17	80	7.42E-04	1.62E-02
R-HSA-2559583	Cellular Senescence	28	163	7.73E-04	1.66E-02
GO:0043542	endothelial cell migration	9	29	7.84E-04	1.67E-02
R-HSA-2980766	Nuclear Envelope Breakdown	12	47	7.99E-04	1.69E-02
R-HSA-1474228	Degradation of the extracellular matrix	25	140	8.12E-04	1.71E-02
GO:0032467	positive regulation of cytokinesis	10	35	8.37E-04	1.74E-02
R-HSA-3560782	Diseases associated with glycosaminoglycan metabolism	11	41	8.36E-04	1.74E-02
R-HSA-6804116	TP53 Regulates Transcription of Genes Involved in G1 Cell Cycle Arrest	6	14	8.93E-04	1.80E-02
GO:0071353	cellular response to interleukin-4	8	24	9.04E-04	1.81E-02
R-HSA-8866652	Synthesis of active ubiquitin: roles of E1 and E2 enzymes	9	30	1.03E-03	2.05E-02
R-HSA-74160	Gene expression (Transcription)	156	1353	1.03E-03	2.05E-02
GO:0030071	regulation of mitotic metaphase/anaphase transition	5	10	1.06E-03	2.07E-02
GO:0071168	protein localization to chromatin	5	10	1.06E-03	2.07E-02

R-HSA-8856688	Golgi-to-ER retrograde transport	24	135	1.09E-03	2.12E-02
GO:0006334	nucleosome assembly	18	90	1.11E-03	2.14E-02
R-HSA-69052	Switching of origins to a post-replicative state	18	90	1.11E-03	2.14E-02
GO:0008284	positive regulation of cell proliferation	64	478	1.12E-03	2.15E-02
GO:0016925	protein sumoylation	16	76	1.17E-03	2.21E-02
R-HSA-6798695	Neutrophil degranulation	64	479	1.18E-03	2.24E-02
GO:0032967	positive regulation of collagen biosynthetic process	8	25	1.22E-03	2.30E-02
R-HSA-76002	Platelet activation, signaling and aggregation	39	260	1.32E-03	2.45E-02
GO:0043312	neutrophil degranulation	64	481	1.31E-03	2.45E-02
R-HSA-157579	Telomere Maintenance	14	63	1.34E-03	2.48E-02
GO:0033146	regulation of intracellular estrogen receptor signaling pathway	6	15	1.37E-03	2.51E-02
R-HSA-2022870	Chondroitin sulfate biosynthesis	7	20	1.37E-03	2.51E-02
R-HSA-8957275	Post-translational protein phosphorylation	20	107	1.47E-03	2.66E-02
GO:0051260	protein homooligomerization	32	203	1.53E-03	2.76E-02
GO:0007077	mitotic nuclear envelope disassembly	11	44	1.57E-03	2.78E-02
R-HSA-68949	Orc1 removal from chromatin	15	71	1.58E-03	2.78E-02
R-HSA-1169408	ISG15 antiviral mechanism	15	71	1.58E-03	2.78E-02
R-HSA-1169410	Antiviral mechanism by IFN-stimulated genes	15	71	1.58E-03	2.78E-02
R-HSA-69473	G2/M DNA damage checkpoint	16	78	1.56E-03	2.78E-02
GO:0001525	angiogenesis	35	229	1.64E-03	2.87E-02
GO:0006024	glycosaminoglycan biosynthetic process	10	38	1.68E-03	2.89E-02
GO:0001837	epithelial to mesenchymal transition	10	38	1.68E-03	2.89E-02
R-HSA-2129379	Molecules associated with elastic fibres	10	38	1.68E-03	2.89E-02
R-HSA-5693568	Resolution of D-loop Structures through Holliday Junction Intermediates	9	32	1.71E-03	2.92E-02
R-HSA-73728	RNA Polymerase I Promoter Opening	9	32	1.71E-03	2.92E-02
R-HSA-174143	APC/C-mediated degradation of cell cycle proteins	17	86	1.73E-03	2.92E-02
R-HSA-453276	Regulation of mitotic cell cycle	17	86	1.73E-03	2.92E-02
R-HSA-6811434	COPI-dependent Golgi-to-ER retrograde traffic	19	101	1.76E-03	2.96E-02
GO:0001701	in utero embryonic development	28	172	1.80E-03	3.01E-02
GO:0042127	regulation of cell proliferation	31	197	1.84E-03	3.07E-02
R-HSA-9006934	Signaling by Receptor Tyrosine Kinases	58	434	1.95E-03	3.17E-02
R-HSA-8939211	ESR-mediated signaling	22	125	1.97E-03	3.18E-02
GO:0031145	anaphase-promoting complex-dependent catabolic process	16	80	2.05E-03	3.31E-02
R-HSA-3700989	Transcriptional Regulation by TP53	50	363	2.06E-03	3.31E-02
GO:0032465	regulation of cytokinesis	10	39	2.07E-03	3.31E-02
GO:0050690	regulation of defense response to virus by virus	8	27	2.13E-03	3.37E-02
R-HSA-5250924	B-WICH complex positively regulates rRNA expression	13	59	2.13E-03	3.37E-02
R-HSA-5693537	Resolution of D-Loop Structures	9	33	2.16E-03	3.39E-02
R-HSA-73857	RNA Polymerase II Transcription	140	1219	2.16E-03	3.39E-02
R-HSA-5693607	Processing of DNA double-strand break ends	16	81	2.35E-03	3.66E-02
R-HSA-9018519	Estrogen-dependent gene expression	21	119	2.38E-03	3.69E-02

R-HSA-3214815	HDACs deacetylate histones	13	60	2.50E-03	3.86E-02
R-HSA-168253	Host Interactions with Influenza Factors	10	40	2.54E-03	3.91E-02
R-HSA-113510	E2F mediated regulation of DNA replication	7	22	2.57E-03	3.93E-02
GO:0043029	T cell homeostasis	8	28	2.75E-03	4.13E-02
R-HSA-164952	The role of Nef in HIV-1 replication and disease pathogenesis	8	28	2.75E-03	4.13E-02
R-HSA-427413	NoRC negatively regulates rRNA expression	15	75	2.79E-03	4.13E-02
GO:1902287	semaphorin-plexin signaling pathway involved in axon guidance	5	12	2.86E-03	4.15E-02
GO:0040007	growth	5	12	2.86E-03	4.15E-02
R-HSA-8866427	VLDLR internalisation and degradation	5	12	2.86E-03	4.15E-02
GO:0001568	blood vessel development	10	41	3.09E-03	4.47E-02
GO:0097190	apoptotic signaling pathway	15	76	3.19E-03	4.59E-02
GO:0006306	DNA methylation	7	23	3.40E-03	4.83E-02
GO:0006302	double-strand break repair	13	62	3.39E-03	4.83E-02
R-HSA-8939236	RUNX1 regulates transcription of genes involved in differentiation of HSCs	18	99	3.38E-03	4.83E-02
GO:0021915	neural tube development	8	29	3.50E-03	4.95E-02
GO:0016477	cell migration	32	214	3.57E-03	5.00E-02

Table S5. Enriched Reactome pathways and GOBP terms in regulated proteins.

term	description	intersect.size	n.pw	pval	qval
GO:0000398	mRNA splicing, via spliceosome	27	244	0.00E+00	0.00E+00
R-HSA-72163	mRNA Splicing - Major Pathway	22	180	4.88E-15	2.21E-12
R-HSA-72172	mRNA Splicing	22	188	1.21E-14	3.65E-12
R-HSA-72203	Processing of Capped Intron-Containing Pre-mRNA	23	238	1.86E-13	4.21E-11
R-HSA-1430728	Metabolism	66	2066	1.63E-11	2.96E-09
GO:0043312	neutrophil degranulation	28	481	9.82E-11	1.27E-08
R-HSA-6798695	Neutrophil degranulation	28	479	8.92E-11	1.27E-08
GO:0002218	activation of innate immune response	8	20	1.14E-10	1.29E-08
R-HSA-8953854	Metabolism of RNA	32	663	4.93E-10	4.96E-08
GO:0032508	DNA duplex unwinding	9	43	5.18E-09	4.68E-07
GO:1904874	positive regulation of telomerase RNA localization to Cajal body	6	15	2.69E-08	2.21E-06
GO:0016070	RNA metabolic process	8	46	1.74E-07	1.31E-05
R-HSA-168249	Innate Immune System	36	1129	1.57E-06	1.09E-04
GO:0042752	regulation of circadian rhythm	7	48	3.61E-06	2.25E-04
R-HSA-1643685	Disease	33	1026	3.75E-06	2.25E-04
GO:0032212	positive regulation of telomere maintenance via telomerase	6	34	5.81E-06	3.10E-04
R-HSA-556833	Metabolism of lipids	26	733	8.13E-06	3.87E-04
R-HSA-168256	Immune System	51	2065	1.59E-05	6.85E-04
GO:0008380	RNA splicing	11	169	2.05E-05	8.45E-04
GO:0006397	mRNA processing	11	172	2.42E-05	8.75E-04
R-HSA-6803529	FGFR2 alternative splicing	5	26	2.33E-05	8.75E-04
R-HSA-3371556	Cellular response to heat stress	8	88	2.63E-05	9.14E-04
GO:0006986	response to unfolded protein	6	50	5.73E-05	1.62E-03
GO:0048146	positive regulation of fibroblast proliferation	6	50	5.73E-05	1.62E-03
GO:0050821	protein stabilization	10	161	7.26E-05	1.93E-03
GO:0001649	osteoblast differentiation	8	101	7.10E-05	1.93E-03
GO:0051973	positive regulation of telomerase activity	5	34	9.00E-05	2.14E-03
GO:0048511	rhythmic process	6	54	8.90E-05	2.14E-03

R-HSA-6785807	Interleukin-4 and 13 signaling	8	111	1.38E-04	3.12E-03
GO:0006695	cholesterol biosynthetic process	5	41	2.24E-04	4.61E-03
GO:0006457	protein folding	10	190	2.82E-04	5.40E-03
GO:0010501	RNA secondary structure unwinding	5	46	3.88E-04	7.03E-03
R-HSA-1660662	Glycosphingolipid metabolism	5	46	3.88E-04	7.03E-03
GO:0006687	glycosphingolipid metabolic process	5	48	4.75E-04	8.26E-03
GO:0006310	DNA recombination	6	73	4.73E-04	8.26E-03
GO:0055114	oxidation-reduction process	18	547	5.18E-04	8.54E-03
GO:1900034	regulation of cellular response to heat	6	76	5.87E-04	8.71E-03
GO:0032481	positive regulation of type I interferon production	5	50	5.74E-04	8.71E-03
R-HSA-5663205	Infectious disease	14	372	5.99E-04	8.71E-03
GO:0006396	RNA processing	6	77	6.30E-04	8.91E-03
GO:0043086	negative regulation of catalytic activity	5	53	7.53E-04	1.02E-02
R-HSA-1280215	Cytokine Signaling in Immune system	20	672	9.02E-04	1.18E-02
R-HSA-211859	Biological oxidations	10	221	9.19E-04	1.19E-02
R-HSA-2262752	Cellular responses to stress	14	393	1.02E-03	1.28E-02
R-HSA-447115	Interleukin-12 family signaling	5	57	1.05E-03	1.29E-02
R-HSA-71387	Metabolism of carbohydrates	11	268	1.15E-03	1.33E-02
R-HSA-162906	HIV Infection	10	229	1.20E-03	1.36E-02
R-HSA-9018519	Estrogen-dependent gene expression	7	119	1.22E-03	1.36E-02
R-HSA-428157	Sphingolipid metabolism	6	88	1.27E-03	1.40E-02
R-HSA-449147	Signaling by Interleukins	15	451	1.35E-03	1.47E-02
R-HSA-73886	Chromosome Maintenance	6	90	1.43E-03	1.54E-02
GO:1990830	cellular response to leukemia inhibitory factor	6	91	1.51E-03	1.61E-02
GO:0007584	response to nutrient	5	62	1.54E-03	1.62E-02
GO:0031647	regulation of protein stability	5	63	1.65E-03	1.67E-02
R-HSA-8939211	ESR-mediated signaling	7	125	1.62E-03	1.67E-02
R-HSA-5654738	Signaling by FGFR2	5	67	2.17E-03	1.97E-02
R-HSA-3371453	Regulation of HSF1-mediated heat shock response	5	68	2.32E-03	2.06E-02
GO:0051259	protein complex oligomerization	5	69	2.47E-03	2.11E-02
R-HSA-1834949	Cytosolic sensors of pathogen-associated DNA	5	69	2.47E-03	2.11E-02
R-HSA-5668914	Diseases of metabolism	6	105	3.12E-03	2.50E-02
R-HSA-3781865	Diseases of glycosylation	7	141	3.19E-03	2.51E-02
GO:0009615	response to virus	6	106	3.27E-03	2.51E-02
R-HSA-211945	Phase I - Functionalization of compounds	6	106	3.27E-03	2.51E-02
GO:0071456	cellular response to hypoxia	6	107	3.43E-03	2.61E-02
R-HSA-190236	Signaling by FGFR	5	78	4.21E-03	2.86E-02
R-HSA-162587	HIV Life Cycle	7	149	4.32E-03	2.92E-02
GO:0022617	extracellular matrix disassembly	5	80	4.69E-03	3.01E-02
GO:0008543	fibroblast growth factor receptor signaling pathway	5	82	5.21E-03	3.14E-02
R-HSA-8953897	Cellular responses to external stimuli	14	470	5.18E-03	3.14E-02
GO:0001666	response to hypoxia	7	161	6.56E-03	3.65E-02
GO:0071356	cellular response to tumor necrosis factor	6	123	6.74E-03	3.70E-02
GO:0072659	protein localization to plasma membrane	6	127	7.85E-03	4.06E-02
GO:0006805	xenobiotic metabolic process	5	91	8.05E-03	4.11E-02
R-HSA-1474244	Extracellular matrix organization	10	300	8.19E-03	4.15E-02
R-HSA-9006931	Signaling by Nuclear Receptors	7	168	8.21E-03	4.15E-02
GO:0006281	DNA repair	8	211	8.40E-03	4.22E-02
R-HSA-3700989	Transcriptional Regulation by TP53	11	363	1.12E-02	5.00E-02
R-HSA-15869	Metabolism of nucleotides	5	97	1.04E-02	5.00E-02

Table S6. Confident LR pairs (179) mirroring TME associated SDC regulated pathways and their clinical relevance.

Ligand : Ligand transcript ; **Receptor :** Receptor transcript ; **Ther.target (Ligand) :** potential target used in clinical trial* (examples are given in brackets) ; **Ther.target (Receptor) :** potential target used in clinical trial* (examples are given in brackets) ; **corr** : Spearman correlation between ligand and receptor ; **qval** : correlation adjused p-value (after Benjamini-Hochberg correction) ; **pval** : correlation p-value ; **num.cor.pw** : number of target genes that are correlated (r>0.5) with the receptor of the LR pair in each pathway ; **pwid** : Reactome and Gene Ontology terms ID ; **L.ct :** Cell types expressing the ligand transcript of the LR pair **; R.ct :** Cell types expressing the receptor transcript of the LR pair. Clinical trials references were retrieved from https://clinicaltrials.gov website.

Ligand	Receptor	Ther. target (Ligand)	Ther. target (Receptor)	pwid	L.ct	R.ct
ADAM9	ITGB1	NA	NA	R-HSA-1566977|R-HSA-202733|R-HSA-6785807	NA	NA
ALOX5AP	ALOX5	NA	Zileuton (NCT01130688)	R-HSA-6785807	Monocytic lineage	T cells;Monocytic lineage
ANGPT1	ITGB1	Trabananib (NCT01664182, NCT01609790)	NA	R-HSA-1566977|R-HSA-202733|R-HSA-6785807	NA	NA
ANGPTL1	TEK	NA	Regorafenib (NCT02736305)	R-HSA-202733	Endothelial cells	Endothelial cells
ANXA1	FPR1	NA	NA	R-HSA-6783783	NA	Monocytic lineage;Neutrophils
B2M	CD3D	NA	NA	R-HSA-198933|R-HSA-202403|R-HSA-202424|R-HSA-202427|R-HSA-202430|R-HSA-202433|R-HSA-388841|R-HSA-389948|R-HSA-8856825|R-HSA-8856828	T cells;NK cells;Monocytic lineage	T cells;Cytotoxic lymphocytes;B lineage;NK cells;Monocytic lineage;Myeloid dendritic cells
B2M	CD3G	NA	NA	R-HSA-202403|R-HSA-202424|R-HSA-202427|R-HSA-202430|R-HSA-202433|R-HSA-2029480|R-HSA-2029482|R-HSA-388841|R-HSA-389948|R-HSA-8856825|R-HSA-8856828	T cells;NK cells;Monocytic lineage	T cells;Cytotoxic lymphocytes;B lineage;NK cells;Monocytic lineage
B2M	HLA-F	NA	NA	R-HSA-1236977|R-HSA-877300|R-HSA-909733|R-HSA-983170	T cells;NK cells;Monocytic lineage	T cells;Cytotoxic lymphocytes;NK cells;Monocytic lineage
B2M	LILRB1	NA	NA	R-HSA-198933	T cells;NK cells;Monocytic lineage	T cells;Monocytic lineage
B2M	LILRB2	NA	NA	R-HSA-198933	T cells;NK cells;Monocytic lineage	T cells;Monocytic lineage
BTLA	CD79A	NA	NA	R-HSA-983695|R-HSA-983705	T cells;Cytotoxic lymphocytes;B lineage	T cells;Cytotoxic lymphocytes;B lineage;Endothelial cells

C3	ADRA2A	NA	NA	R-HSA-163685	T cells;B lineage;Endothelial cells	NA
C3	CD19	NA	NA	R-HSA-199418|R-HSA-2219528|R-HSA-6811558|R-HSA-983695|R-HSA-983705	T cells;B lineage;Endothelial cells	T cells;Cytotoxic lymphocytes;B lineage;NK cells;Endothelial cells
CCL11	CCR5	NA	Maraviroc (NCT01736813); Leronlimab (NCT03838367); Vicriviroc (NCT03631407)	R-HSA-6783783	T cells;Monocytic lineage	T cells;Cytotoxic lymphocytes;Monocytic lineage;Myeloid dendritic cells
CCL13	CCR1	NA	NA	R-HSA-6783783	T cells;Monocytic lineage;Myeloid dendritic cells;Neutrophils	T cells;Monocytic lineage
CCL13	CCR2	NA	MLN1202	R-HSA-6783783	T cells;Monocytic lineage;Myeloid dendritic cells;Neutrophils	T cells;Cytotoxic lymphocytes;B lineage;NK cells;Monocytic lineage;Myeloid dendritic cells
CCL13	CCR5	NA	Maraviroc (NCT01736813); Leronlimab (NCT03838367); Vicriviroc (NCT03631407)	R-HSA-6783783	T cells;Monocytic lineage;Myeloid dendritic cells;Neutrophils	T cells;Cytotoxic lymphocytes;Monocytic lineage;Myeloid dendritic cells
CCL23	CCR1	NA	NA	R-HSA-6783783	Monocytic lineage;Neutrophils	T cells;Monocytic lineage
CCL24	CCR2	NA	MLN1202	R-HSA-6783783	NA	T cells;Cytotoxic lymphocytes;B lineage;NK cells;Monocytic lineage;Myeloid dendritic cells
CCL3	CCR1	NA	NA	R-HSA-6783783	Monocytic lineage	T cells;Monocytic lineage
CCL3	CCR5	NA	Maraviroc (NCT01736813); Leronlimab (NCT03838367); Vicriviroc (NCT03631407)	R-HSA-6783783	Monocytic lineage	T cells;Cytotoxic lymphocytes;Monocytic lineage;Myeloid dendritic cells
CCL4	CCR1	NA	NA	R-HSA-6783783	T cells;Monocytic lineage	T cells;Monocytic lineage

CCL4	CCR5	NA	Maraviroc (NCT01736813); Leronlimab (NCT03838367); Vicriviroc (NCT03631407)	R-HSA-6783783	T cells;Monocytic lineage	T cells;Cytotoxic lymphocytes;Monocytic lineage;Myeloid dendritic cells
CCL5	CCR1	NA	NA	R-HSA-6783783	T cells;Cytotoxic lymphocytes;NK cells	T cells;Monocytic lineage
CCL5	CCR5	NA	Maraviroc (NCT01736813); Leronlimab (NCT03838367); Vicriviroc (NCT03631407)	R-HSA-6783783	T cells;Cytotoxic lymphocytes;NK cells	T cells;Cytotoxic lymphocytes;Monocytic lineage;Myeloid dendritic cells
CCL8	CCR1	NA	NA	R-HSA-6783783	NA	T cells;Monocytic lineage
CCL8	CCR5	NA	Maraviroc (NCT01736813); Leronlimab (NCT03838367); Vicriviroc (NCT03631407)	R-HSA-6783783	NA	T cells;Cytotoxic lymphocytes;Monocytic lineage;Myeloid dendritic cells
CD14	ITGB2	NA	NA	R-HSA-166016|R-HSA-168898|R-HSA-198933|R-HSA-202733|R-HSA-6785807	T cells;Monocytic lineage	T cells;Monocytic lineage
CD14	TLR4	NA	GLA-SE (NCT03982121); GSK1795091(NCT02798978);GLA-SE (NCT02180698); GSK1795091(NCT03447314)	R-HSA-109581|R-HSA-166016|R-HSA-166058|R-HSA-168138|R-HSA-168179|R-HSA-168181|R-HSA-168188|R-HSA-168898|R-HSA-181438|R-HSA-5357801|R-HSA-975138|R-HSA-975155	T cells;Monocytic lineage	Monocytic lineage
CD274	PDCD1	Atezolizumab (NCT03087864); Durvalumab (NCT02777710)	Anti-PD1 monoclonal antibody (NCT03983057, NCT03977272);Nivolumab;Pembrolizumab	R-HSA-388841|R-HSA-389948	T cells;Cytotoxic lymphocytes;Monocytic lineage	T cells;Cytotoxic lymphocytes;B lineage;NK cells;Monocytic lineage

CD80	CD28	CD80/86-CAR-T cell immunotherapy	NA	R-HSA-162909|R-HSA-199418|R-HSA-2219528|R-HSA-2219530|R-HSA-388841|R-HSA-389356|R-HSA-389357|R-HSA-389359|R-HSA-6811558	T cells;Monocytic lineage	T cells;Cytotoxic lymphocytes;Monocytic lineage
CD80	CTLA4	CD80/86-CAR-T cell immunotherapy	AGEN1181 (NCT03860272); Tremelimumab (NCT03019003)	R-HSA-388841|R-HSA-8877330	T cells;Monocytic lineage	T cells;Cytotoxic lymphocytes;B lineage;NK cells;Monocytic lineage
CD86	CD28	CD80/86-CAR-T cell immunotherapy	NA	R-HSA-162909|R-HSA-199418|R-HSA-2219528|R-HSA-2219530|R-HSA-388841|R-HSA-389356|R-HSA-389357|R-HSA-389359|R-HSA-6811558	T cells;NK cells;Monocytic lineage;Myeloid dendritic cells;Neutrophils	T cells;Cytotoxic lymphocytes;Monocytic lineage
CD86	CTLA4	CD80/86-CAR-T cell immunotherapy	AGEN1181 (NCT03860272);Tremelimumab (NCT03019003)	R-HSA-388841|R-HSA-8877330	T cells;NK cells;Monocytic lineage;Myeloid dendritic cells;Neutrophils	T cells;Cytotoxic lymphocytes;B lineage;NK cells;Monocytic lineage
COL18A1	ITGA5	endostatin	NA	R-HSA-1566977|R-HSA-202733	Endothelial cells	Endothelial cells
COL18A1	ITGB3	endostatin	NA	R-HSA-194138|R-HSA-202733|R-HSA-4420097|R-HSA-5674135|R-HSA-6802946|R-HSA-6802948|R-HSA-6802949|R-HSA-6802952|R-HSA-6802955|R-HSA-6802957	Endothelial cells	Endothelial cells

COL18A1	KDR	endostatin	apatinib (NCT03587129)	R-HSA-194138|R-HSA-4420097	Endothelial cells	Endothelial cells
COL1A1	ITGA1	NA	NA	R-HSA-397014	Fibroblasts	Endothelial cells
COL1A2	ITGA1	NA	NA	R-HSA-397014	Fibroblasts	Endothelial cells
COL4A1	ITGA1	NA	NA	R-HSA-397014	Endothelial cells	Endothelial cells
COL4A2	ITGB3	NA	NA	R-HSA-194138|R-HSA-202733|R-HSA-4420097|R-HSA-5674135|R-HSA-6802946|R-HSA-6802948|R-HSA-6802949|R-HSA-6802952|R-HSA-6802955|R-HSA-6802957	Endothelial cells	Endothelial cells
COL5A1	ITGA1	NA	NA	R-HSA-397014	Fibroblasts	Endothelial cells
COL5A2	ITGA1	NA	NA	R-HSA-397014	Fibroblasts	Endothelial cells
COL6A1	ITGB1	NA	NA	R-HSA-1566977|R-HSA-202733|R-HSA-6785807	Fibroblasts	NA
COL6A2	ITGB1	NA	NA	R-HSA-1566977|R-HSA-202733|R-HSA-6785807	Fibroblasts	NA
COL6A3	ITGA1	Aldesleukin + utolimumab (NCT03318900)	NA	R-HSA-397014	Fibroblasts	Endothelial cells
COL6A3	ITGB1	Aldesleukin + utolimumab (NCT03318900)	NA	R-HSA-1566977|R-HSA-202733|R-HSA-6785807	Fibroblasts	NA
DLL4	NOTCH4	NOV150101 (NCT03292783)	NA	R-HSA-157118|R-HSA-3781865	Endothelial cells	Endothelial cells
DMP1	ITGB3	NA	NA	R-HSA-194138|R-HSA-202733|R-HSA-4420097|R-HSA-5674135|R-HSA-6802946|R-HSA-6802948|R-HSA-6802949|R-HSA-6802952|R-HSA-6802955|R-HSA-6802957	NA	Endothelial cells
EDIL3	ITGB5	NA	NA	R-HSA-397014	Fibroblasts	NA

EFNB2	PECAM1	sEphB4-HSA with cetuximab and radiation (NCT04091867)	Daratumumab (NCT03734198)	R-HSA-202733|R-HSA-418346	NA	NA
EGF	ADRB2	NA	Carvedilol (NCT02944201)	R-HSA-5689880	NA	NA
F13A1	ITGB1	NA	NA	R-HSA-1566977|R-HSA-202733|R-HSA-6785807	Fibroblasts	NA
FASLG	FAS	NA	NA	R-HSA-109581|R-HSA-5357801	T cells;Cytotoxic lymphocytes;B lineage;NK cells;Monocytic lineage	NK cells;Monocytic lineage
FBN1	ITGB3	NA	NA	R-HSA-194138|R-HSA-202733|R-HSA-4420097|R-HSA-5674135|R-HSA-6802946|R-HSA-6802948|R-HSA-6802949|R-HSA-6802952|R-HSA-6802955|R-HSA-6802957	Endothelial cells;Fibroblasts	Endothelial cells
FN1	ITGA5	NA	NA	R-HSA-1566977|R-HSA-202733	Fibroblasts	Endothelial cells
FN1	ITGB1	NA	NA	R-HSA-1566977|R-HSA-202733|R-HSA-6785807	Fibroblasts	NA
FN1	ITGB3	NA	NA	R-HSA-194138|R-HSA-202733|R-HSA-4420097|R-HSA-5674135|R-HSA-6802946|R-HSA-6802948|R-HSA-6802949|R-HSA-6802952|R-HSA-6802955|R-HSA-6802957	Fibroblasts	Endothelial cells
GNAI2	CCR5	NA	Maraviroc (NCT01736813); Leronlimab (NCT03838367); Vicriviroc (NCT03631407)	R-HSA-6783783	T cells;Monocytic lineage;Endothelial cells	T cells;Cytotoxic lymphocytes;Monocytic lineage;Myeloid dendritic cells

HLA-A	CD3D	NA	NA	R-HSA-198933|R-HSA-202403|R-HSA-202424|R-HSA-202427|R-HSA-202430|R-HSA-202433|R-HSA-388841|R-HSA-389948|R-HSA-8856825|R-HSA-8856828	T cells;Cytotoxic lymphocytes;NK cells;Monocytic lineage	T cells;Cytotoxic lymphocytes;B lineage;NK cells;Monocytic lineage;Myeloid dendritic cells
HLA-A	CD3G	NA	NA	R-HSA-202403|R-HSA-202424|R-HSA-202427|R-HSA-202430|R-HSA-202433|R-HSA-2029480|R-HSA-2029482|R-HSA-388841|R-HSA-389948|R-HSA-8856825|R-HSA-8856828	T cells;Cytotoxic lymphocytes;NK cells;Monocytic lineage	T cells;Cytotoxic lymphocytes;B lineage;NK cells;Monocytic lineage
HLA-A	LILRB1	NA	NA	R-HSA-198933	T cells;Cytotoxic lymphocytes;NK cells;Monocytic lineage	T cells;Monocytic lineage
HLA-A	LILRB2	NA	NA	R-HSA-198933	T cells;Cytotoxic lymphocytes;NK cells;Monocytic lineage	T cells;Monocytic lineage
HLA-B	CD3D	NA	NA	R-HSA-198933|R-HSA-202403|R-HSA-202424|R-HSA-202427|R-HSA-202430|R-HSA-202433|R-HSA-388841|R-HSA-389948|R-HSA-8856825|R-HSA-8856828	T cells;Cytotoxic lymphocytes;NK cells;Monocytic lineage	T cells;Cytotoxic lymphocytes;B lineage;NK cells;Monocytic lineage;Myeloid dendritic cells
HLA-B	CD3G	NA	NA	R-HSA-202403|R-HSA-202424|R-HSA-202427|R-HSA-202430|R-HSA-202433|R-HSA-2029480|R-HSA-2029482|R-HSA-388841|R-HSA-389948|R-HSA-8856825|R-HSA-8856828	T cells;Cytotoxic lymphocytes;NK cells;Monocytic lineage	T cells;Cytotoxic lymphocytes;B lineage;NK cells;Monocytic lineage
HLA-B	KLRD1	NA	NA	R-HSA-2172127|R-HSA-2424491	T cells;Cytotoxic lymphocytes;NK cells;Monocytic lineage	NA
HLA-B	LILRB1	NA	NA	R-HSA-198933	T cells;Cytotoxic lymphocytes;NK cells;Monocytic lineage	T cells;Monocytic lineage

HLA-B	LILRB2	NA	NA	R-HSA-198933	T cells;Cytotoxic lymphocytes;NK cells;Monocytic lineage	T cells;Monocytic lineage
HLA-C	CD3D	NA	NA	R-HSA-198933|R-HSA-202403|R-HSA-202424|R-HSA-202427|R-HSA-202430|R-HSA-202433|R-HSA-388841|R-HSA-389948|R-HSA-8856825|R-HSA-8856828	T cells;Cytotoxic lymphocytes;NK cells;Monocytic lineage	T cells;Cytotoxic lymphocytes;B lineage;NK cells;Monocytic lineage;Myeloid dendritic cells
HLA-C	CD3G	NA	NA	R-HSA-202403|R-HSA-202424|R-HSA-202427|R-HSA-202430|R-HSA-202433|R-HSA-2029480|R-HSA-2029482|R-HSA-388841|R-HSA-389948|R-HSA-8856825|R-HSA-8856828	T cells;Cytotoxic lymphocytes;NK cells;Monocytic lineage	T cells;Cytotoxic lymphocytes;B lineage;NK cells;Monocytic lineage
HLA-C	LILRB1	NA	NA	R-HSA-198933	T cells;Cytotoxic lymphocytes;NK cells;Monocytic lineage	T cells;Monocytic lineage
HLA-C	LILRB2	NA	NA	R-HSA-198933	T cells;Cytotoxic lymphocytes;NK cells;Monocytic lineage	T cells;Monocytic lineage
HLA-E	KLRD1	NA	NA	R-HSA-2172127|R-HSA-2424491	NA	NA
HRAS	SDC2	Tipifarnib (NCT02383927)	NA	R-HSA-3781865|R-HSA-381426|R-HSA-8957275	NA	Fibroblasts
IBSP	ITGB3	NA	NA	R-HSA-194138|R-HSA-202733|R-HSA-4420097|R-HSA-5674135|R-HSA-6802946|R-HSA-6802948|R-HSA-6802949|R-HSA-6802952|R-HSA-6802955|R-HSA-6802957	NA	Endothelial cells
ICAM1	IL2RA	CVA21 (NCT00832559, NCT00636558)	aldesleukin (Proleukin)	R-HSA-392451|R-HSA-397795|R-HSA-451927|R-HSA-512988|R-HSA-9020558|R-HSA-912526	T cells;Cytotoxic lymphocytes;NK cells	T cells;Monocytic lineage

ICAM1	IL2RG	CVA21 (NCT00832559, NCT00636558)	aldesleukin (Proleukin)	R-HSA-1266695|R-HSA-392451|R-HSA-397795|R-HSA-451927|R-HSA-512988|R-HSA-6785807|R-HSA-8983432|R-HSA-9020558|R-HSA-9020958|R-HSA-912526	T cells;Cytotoxic lymphocytes;NK cells	T cells;Cytotoxic lymphocytes;B lineage;NK cells;Monocytic lineage
ICAM1	ITGAM	CVA21 (NCT00832559, NCT00636558)	NA	R-HSA-166016|R-HSA-168898|R-HSA-202733|R-HSA-6785807	T cells;Cytotoxic lymphocytes;NK cells	T cells;Monocytic lineage
ICAM1	ITGAX	CVA21 (NCT00832559, NCT00636558)	NA	R-HSA-202733|R-HSA-6785807	T cells;Cytotoxic lymphocytes;NK cells	T cells;Monocytic lineage
ICAM1	ITGB2	CVA21 (NCT00832559, NCT00636558)	NA	R-HSA-166016|R-HSA-168898|R-HSA-198933|R-HSA-202733|R-HSA-6785807	T cells;Cytotoxic lymphocytes;NK cells	T cells;Monocytic lineage
ICAM3	ITGB2	NA	NA	R-HSA-166016|R-HSA-168898|R-HSA-198933|R-HSA-202733|R-HSA-6785807	T cells;Cytotoxic lymphocytes;B lineage;NK cells;Monocytic lineage	T cells;Monocytic lineage
ICAM4	ITGA4	NA	NA	R-HSA-202733	T cells;Cytotoxic lymphocytes;NK cells	Monocytic lineage
ICAM5	ITGB2	NA	NA	R-HSA-166016|R-HSA-168898|R-HSA-198933|R-HSA-202733|R-HSA-6785807	NA	T cells;Monocytic lineage
IGF2	IGF1R	NA	NA	R-HSA-2404192	NA	Endothelial cells
IL10	IL10RA	NA	NA	R-HSA-6783783	NA	NA
IL10	IL10RB	NA	NA	R-HSA-6783783	NA	NA
IL12B	IL12RB1	bacTRL-IL-12 (NCT04025307) ; NHS-IL-12 (NCT01417546)	NA	R-HSA-447115|R-HSA-9020591	T cells;Myeloid dendritic cells	T cells;Cytotoxic lymphocytes;NK cells;Monocytic lineage
IL15	IL15RA	rhIL-15 (NCT03388632)	NA	R-HSA-451927	T cells;Cytotoxic lymphocytes;NK cells	NA

IL15	IL2RB	rhIL-15 (NCT03388632)	aldesleukin (Proleukin)	R-HSA-392451|R-HSA-397795|R-HSA-451927|R-HSA-512988|R-HSA-9020558	T cells;Cytotoxic lymphocytes;NK cells	T cells;Cytotoxic lymphocytes;B lineage;NK cells
IL15	IL2RG	rhIL-15 (NCT03388632)	aldesleukin (Proleukin)	R-HSA-1266695|R-HSA-392451|R-HSA-397795|R-HSA-451927|R-HSA-512988|R-HSA-6785807|R-HSA-8983432|R-HSA-9020558|R-HSA-9020958|R-HSA-912526	T cells;Cytotoxic lymphocytes;NK cells	T cells;Cytotoxic lymphocytes;B lineage;NK cells;Monocytic lineage
IL16	CCR5	vaccine with gene modified SJNB-JF-Lptn (NCT00703222)	Maraviroc (NCT01736813);Leronlimab (NCT03838367);Vicriviroc (NCT03631407)	R-HSA-6783783	T cells;Cytotoxic lymphocytes;B lineage	T cells;Cytotoxic lymphocytes;Monocytic lineage;Myeloid dendritic cells
IL18	IL18BP	NA	NA	R-HSA-446652	T cells;Monocytic lineage	T cells;Cytotoxic lymphocytes;B lineage;Monocytic lineage
IL18	IL18RAP	NA	NA	R-HSA-446652|R-HSA-9012546	T cells;Monocytic lineage	T cells;Cytotoxic lymphocytes;NK cells
IL1A	IL1R2	NA	CAN04 (NCT0326731)	R-HSA-446652|R-HSA-6783783	NA	NA
IL1RN	IL1R2	NA	NA	R-HSA-446652|R-HSA-6783783	NA	NA
IL21	IL21R	recombinant interleukin-21 (NCT00514085)	NA	R-HSA-451927|R-HSA-9020958	T cells;Cytotoxic lymphocytes;Monocytic lineage	T cells;Cytotoxic lymphocytes;B lineage;NK cells;Monocytic lineage
IL21	IL2RG	recombinant interleukin-21 (NCT00514085)	NA	R-HSA-1266695|R-HSA-392451|R-HSA-397795|R-HSA-451927|R-HSA-512988|R-HSA-6785807|R-HSA-8983432|R-HSA-9020558|R-HSA-9020958|R-HSA-912526	T cells;Cytotoxic lymphocytes;Monocytic lineage	T cells;Cytotoxic lymphocytes;B lineage;NK cells;Monocytic lineage

IL2	IL2RB	aldesleukin (Proleukin)	BNZ132-1-40 (NCT03239392)	R-HSA-392451|R-HSA-397795|R-HSA-451927|R-HSA-512988|R-HSA-9020558	T cells	T cells;Cytotoxic lymphocytes;B lineage;NK cells
IL2	IL2RG	aldesleukin (Proleukin)	BNZ132-1-40 (NCT03239392)	R-HSA-1266695|R-HSA-392451|R-HSA-397795|R-HSA-451927|R-HSA-512988|R-HSA-6785807|R-HSA-8983432|R-HSA-9020558|R-HSA-9020958|R-HSA-912526	T cells	T cells;Cytotoxic lymphocytes;B lineage;NK cells;Monocytic lineage
JAG1	NOTCH3	Gamma-Secretase Inhibitor RO4929097 (NCT01175343)	NA	R-HSA-157118|R-HSA-3781865|R-HSA-9012852|R-HSA-9013508	Endothelial cells	Endothelial cells
JAG1	NOTCH4	Gamma-Secretase Inhibitor RO4929097 (NCT01175343)	NA	R-HSA-157118|R-HSA-3781865	Endothelial cells	Endothelial cells
JAM3	ITGB1	NA	NA	R-HSA-1566977|R-HSA-202733|R-HSA-6785807	NA	NA
LAMA4	ITGB1	NA	NA	R-HSA-1566977|R-HSA-202733|R-HSA-6785807	Endothelial cells	NA
LAMB1	ITGA1	NA	NA	R-HSA-397014	Fibroblasts	Endothelial cells
LAMB1	ITGB1	NA	NA	R-HSA-1566977|R-HSA-202733|R-HSA-6785807	Fibroblasts	NA
LAMB3	COL17A1	NA	NA	R-HSA-1474228|R-HSA-1474290|R-HSA-1650814|R-HSA-2022090	NA	NA
LAMB3	ITGA6	NA	NA	R-HSA-1474290	NA	NA
LAMC1	ITGA1	NA	NA	R-HSA-397014	Endothelial cells	Endothelial cells
LAMC1	ITGB1	NA	NA	R-HSA-1566977|R-HSA-202733|R-HSA-6785807	Endothelial cells	NA
LAMC2	COL17A1	NA	NA	R-HSA-1474228|R-HSA-1474290|R-HSA-1650814|R-HSA-2022090	NA	NA
LAMC2	ITGA6	NA	NA	R-HSA-1474290	NA	NA

LGALS9	HAVCR2	Galectin Inhibitor (GR-MD-02) (NCT02117362)	anti-TIM-3 antibody TSR-022 (NCT02817633);LY3321367 (NCT03099109);BGB-A425 (NCT03744468)	R-HSA-451927	T cells;NK cells;Monocytic lineage	T cells;Monocytic lineage
LTA	TNFRSF14	NA	NA	R-HSA-388841|R-HSA-5668541	T cells;Cytotoxic lymphocytes;B lineage;NK cells;Monocytic lineage	T cells;Monocytic lineage
LTA	TNFRSF1B	NA	NA	R-HSA-5668541|R-HSA-6783783|R-HSA-6785807	T cells;Cytotoxic lymphocytes;B lineage;NK cells;Monocytic lineage	T cells;Cytotoxic lymphocytes;NK cells;Monocytic lineage
LTBP3	ITGB5	NA	NA	R-HSA-397014	Endothelial cells	NA
LY96	TLR4	NA	TLR4 agonist GLA-SE (NCT03982121)	R-HSA-109581|R-HSA-166016|R-HSA-166058|R-HSA-168138|R-HSA-168179|R-HSA-168181|R-HSA-168188|R-HSA-168898|R-HSA-181438|R-HSA-5357801|R-HSA-975138|R-HSA-975155	T cells;Monocytic lineage	Monocytic lineage
MMP2	SDC2	COX2 inhibtors (celecoxib) (NCT00653250), metalloproteinase inhibitor (marimastat) (NCT00003011)	NA	R-HSA-3781865|R-HSA-381426|R-HSA-8957275	Fibroblasts	Fibroblasts
MMP9	ITGAM	COX2 inhibtors (celecoxib) (NCT00653250), metalloproteinase inhibitor (marimastat) (NCT00003011)	NA	R-HSA-166016|R-HSA-168898|R-HSA-202733|R-HSA-6785807	NA	T cells;Monocytic lineage
MYOC	FZD7	NA	NA	R-HSA-3858494	NA	NA
NID1	ITGB1	NA	NA	R-HSA-1566977|R-HSA-202733|R-HSA-6785807	Fibroblasts	NA

NID1	ITGB3	NA	NA	R-HSA-194138|R-HSA-202733|R-HSA-4420097|R-HSA-5674135|R-HSA-6802946|R-HSA-6802948|R-HSA-6802949|R-HSA-6802952|R-HSA-6802955|R-HSA-6802957	Fibroblasts	Endothelial cells
PDGFB	PDGFRB	TKI258 (Dovitinib) (NCT01753713)	BAY 43-9006 (NCT0009545); Anlotinib Hydrochloride (NCT04042597);Dasatinib (NCT03297606)	R-HSA-199418|R-HSA-2219528|R-HSA-2219530|R-HSA-6811558	Endothelial cells	Endothelial cells;Fibroblasts
PDGFD	PDGFRA	Anlotinib (NCT03672136)	Olaratumab (NCT01204710)	R-HSA-199418|R-HSA-2219528|R-HSA-2219530|R-HSA-6811558	NA	NA
PECAM1	ITGB3	Daratumumab (NCT03734198)	NA	R-HSA-194138|R-HSA-202733|R-HSA-4420097|R-HSA-5674135|R-HSA-6802946|R-HSA-6802948|R-HSA-6802949|R-HSA-6802952|R-HSA-6802955|R-HSA-6802957	Endothelial cells	Endothelial cells
PGF	FLT1	Ziv-Aflibercept (NCT02192541)	VEGFR1-1084 (NCT00655785)	R-HSA-194138	Endothelial cells	Endothelial cells
PGF	NRP1	Ziv-Aflibercept (NCT02192541)	NA	R-HSA-194138	Endothelial cells	NA
PLAU	ITGB5	NA	NA	R-HSA-397014	Fibroblasts	NA
SELPLG	ITGAM	NA	NA	R-HSA-166016|R-HSA-168898|R-HSA-202733|R-HSA-6785807	T cells;Cytotoxic lymphocytes;Monocytic lineage	T cells;Monocytic lineage
SELPLG	ITGB2	NA	NA	R-HSA-166016|R-HSA-168898|R-HSA-198933|R-HSA-202733|R-HSA-6785807	T cells;Cytotoxic lymphocytes;Monocytic lineage	T cells;Monocytic lineage
SEMA3A	NRP1	NA	NA	R-HSA-194138	NA	NA
SERPINC1	SDC2	NA	NA	R-HSA-3781865|R-HSA-381426|R-HSA-8957275	NA	Fibroblasts

TCTN1	TMEM67	NA	NA	R-HSA-5620912	NA	NA
TGFB1	ITGB3	TGF-β Receptor Inhibitor LY2157299 (NCT02452008); AVID200 (NCT03834662)	NA	R-HSA-194138|R-HSA-202733|R-HSA-4420097|R-HSA-5674135|R-HSA-6802946|R-HSA-6802948|R-HSA-6802949|R-HSA-6802952|R-HSA-6802955|R-HSA-6802957	Endothelial cells	Endothelial cells
TGFB3	ITGB5	AVID200 (NCT03895112)	NA	R-HSA-397014	Endothelial cells	NA
TLN1	ITGB3	NA	NA	R-HSA-194138|R-HSA-202733|R-HSA-4420097|R-HSA-5674135|R-HSA-6802946|R-HSA-6802948|R-HSA-6802949|R-HSA-6802952|R-HSA-6802955|R-HSA-6802957	Endothelial cells	Endothelial cells
TNFSF13B	TNFRSF13B	NA	NA	R-HSA-5668541	T cells;Cytotoxic lymphocytes;NK cells;Monocytic lineage	T cells;B lineage;Endothelial cells
TNFSF4	TNFRSF4	SL-279252 (PD1-Fc-OX40L) (NCT03894618)	anti-OX40 (MEDI6469) (NCT02559024)	R-HSA-5668541	Fibroblasts	T cells;Cytotoxic lymphocytes;Monocytic lineage
TNF	TNFRSF1B	Tumor-targeting Human L19TNFα Monoclonal Antibody-cytokine Fusion Protein (NCT02076620)	NA	R-HSA-5668541|R-HSA-6783783|R-HSA-6785807	T cells	T cells;Cytotoxic lymphocytes;NK cells;Monocytic lineage
VCAM1	ITGA4	NA	NA	R-HSA-202733	T cells	Monocytic lineage
VCAM1	ITGB2	NA	NA	R-HSA-166016|R-HSA-168898|R-HSA-198933|R-HSA-	T cells	T cells;Monocytic lineage

				202733|R-HSA-6785807		
VEGFC	FLT1	VGX-100 (NCT01514123)	VEGFR1-1084 (NCT00655785)	R-HSA-194138	NA	Endothelial cells
VWF	ITGB3	NA	NA	R-HSA-194138|R-HSA-202733|R-HSA-4420097|R-HSA-5674135|R-HSA-6802946|R-HSA-6802948|R-HSA-6802949|R-HSA-6802952|R-HSA-6802955|R-HSA-6802957	Endothelial cells	Endothelial cells
WNT3	FZD7	NA	NA	R-HSA-3858494	NA	NA
WNT3	LRP6	NA	NA	R-HSA-4791275	NA	NA
WNT5A	FZD1	NA	NA	R-HSA-3858494|R-HSA-4086400	NA	NA
LGALS1	ITGB1	GR-MD-02 (NCT02117362)	NA	R-HSA-1566977|R-HSA-202733|R-HSA-6785807	NA	NA
DLL4	NOTCH3	NOV1501 (ABL001) (NCT03292783)	NA	R-HSA-157118|R-HSA-3781865|R-HSA-9012852|R-HSA-9013508	Endothelial cells	Endothelial cells
PTPRC	CD22	NA	NA	R-HSA-983695|R-HSA-983705	NA	NA
CD1D	LILRB2	NA	NA	R-HSA-198933	NA	T cells;Monocytic lineage
CD274	CD80	Durvalumab (NCT02484404)	NA	R-HSA-199418|R-HSA-2219528|R-HSA-2219530|R-HSA-388841|R-HSA-389356|R-HSA-389357|R-HSA-389359|R-HSA-389513|R-HSA-6783783|R-HSA-6811558	T cells;Cytotoxic lymphocytes;Monocytic lineage	NA
CD177	PECAM1	NA	Daratumumab (NCT03734198)	R-HSA-202733|R-HSA-418346	NA	NA
B2M	CD1B	NA	NA	GO:0002250	T cells;NK cells;Monocytic lineage	T cells;Monocytic lineage;Myeloid dendritic cells

B2M	CD247	NA	NA	GO:0002250|GO:0031295|GO:0038096|GO:0050690|GO:0050852	T cells;NK cells;Monocytic lineage	T cells;Cytotoxic lymphocytes;B lineage;NK cells;Monocytic lineage;Myeloid dendritic cells
BTLA	CD247	NA	NA	GO:0002250|GO:0031295|GO:0038096|GO:0050690|GO:0050852	T cells;Cytotoxic lymphocytes;B lineage	T cells;Cytotoxic lymphocytes;B lineage;NK cells;Monocytic lineage;Myeloid dendritic cells
CD40LG	CD40	NA	Fc-engineered Anti-CD40 Monoclonal Antibody (2141-V11) (NCT04059588)	GO:0033209|GO:0043123	T cells;Cytotoxic lymphocytes;B lineage;NK cells;Monocytic lineage	T cells;B lineage;Monocytic lineage
COL1A1	ITGA11	NA	NA	GO:0030198	Fibroblasts	Fibroblasts
COL1A1	ITGAV	NA	Cilengitide (EMD121974) (NCT01122888)	GO:0030198	Fibroblasts	Fibroblasts
COL1A2	ITGA11	NA	NA	GO:0030198	Fibroblasts	Fibroblasts
COL1A2	ITGAV	NA	Cilengitide (EMD121974) (NCT01122888)	GO:0030198	Fibroblasts	Fibroblasts
CSF1	CSF1R	NA	PEXIDARTINIB (NCT02777710);Chiauranib (NCT03216343)	GO:0030097	NA	Monocytic lineage;Neutrophils
EFNA1	EPHA10	NA	NA	GO:0043410	NA	NA
FBN1	ITGAV	NA	Cilengitide (EMD121974) (NCT01122888)	GO:0030198	Endothelial cells;Fibroblasts	Fibroblasts
FGF17	FGFR3	NA	Dovitinib (NCT01732107)	GO:0046854|GO:0051897	NA	NA
FGF2	FGFR1	Tinzaparin	Rogaratinib (NCT04040725)	GO:0051897	NA	NA
FGF2	FGFR2	Tinzaparin	BAY1187982 (NCT02368951)	GO:0046854|GO:0051897	NA	NA
FGF7	FGFR2	NA	BAY1187982 (NCT02368951)	GO:0046854|GO:0051897	NA	NA
FN1	ITGAV	NA	Cilengitide (EMD121974) (NCT01122888)	GO:0030198	Fibroblasts	Fibroblasts

LAMA5	ITGA3	NA	NA	GO:0030198	NA	NA
LAMB3	ITGA3	NA	NA	GO:0030198	NA	NA
LAMC2	ITGA3	NA	NA	GO:0030198	NA	NA
LTB	CD40	NA	Fc-engineered Anti-CD40 Monoclonal Antibody (2141-V11) (NCT04059588)	GO:0033209|GO:0043123	T cells;Cytotoxic lymphocytes;B lineage;NK cells;Monocytic lineage;Myeloid dendritic cells	T cells;B lineage;Monocytic lineage
NCAM1	FGFR1	Lorvotuzumab Mertansine (NCT02452554)	Rogaratinib (NCT04040725)	GO:0051897	Fibroblasts	NA
NID1	ITGAV	NA	Cilengitide (EMD121974) (NCT01122888)	GO:0030198	Fibroblasts	Fibroblasts
NRG4	ERBB4	NA	Seribantumab (NCT03241810) (NCT02387216), Patritumab (NCT02633800)	GO:0046854|GO:0051897	NA	Neutrophils
PLAU	ITGAV	NA	Cilengitide (EMD121974) (NCT01122888)	GO:0030198	Fibroblasts	Fibroblasts
PSAP	CD1B	NA	NA	GO:0002250	T cells;Monocytic lineage	T cells;Monocytic lineage;Myeloid dendritic cells
TGFB1	TGFBR2	NA	TGF-β Receptor Inhibitor LY2157299 (NCT02452008);AVID200 (NCT03834662)	GO:0007219	Endothelial cells	Endothelial cells
TGFB3	TGFBR2	AVID200 (NCT03834662)	TGF-β Receptor Inhibitor LY2157299 (NCT02452008);AVID200 (NCT03834662)	GO:0007219	Endothelial cells	Endothelial cells
JAM2	JAM3	NA	NA	GO:0030198	NA	NA

Supplementary References

1. Zhao S, Zhang Y, Gamini R, Zhang B, von Schack D. Evaluation of two main RNA-seq approaches for gene quantification in clinical RNA sequencing: polyA+ selection versus rRNA depletion. Sci Rep [Internet]. 2018 [cited 15 September 2019]; 8. Available at: https://www.ncbi.nlm.nih.gov/pmc/articles/PMC5859127/
